# Higher growth of the apple (*Malus* × *domestica* Borkh.) fruit cortex is supported by resource intensive metabolism during early development

**DOI:** 10.1186/s12870-020-2280-2

**Published:** 2020-02-13

**Authors:** Shan Jing, Anish Malladi

**Affiliations:** Department of Horticulture, University of Georgia, 1111 Miller Plant Sciences, Athens, GA 30602 USA

**Keywords:** Carbon metabolism, Cell production, Fruit growth and development, Fruit load reduction, Metabolic profiling, Nitrogen metabolism, Sink activity

## Abstract

**Background:**

The major fleshy tissues of the apple fruit are spatially separable into cortex and pith. These tissues display differential growth during development. Key features of such differential growth, and sink metabolic programs supporting it have not been investigated previously. We hypothesized that differential growth between these fruit tissues is supported by differential sink metabolic programs, particularly during early development. Growth, metabolite concentrations, and transcript abundance of metabolism-related genes were measured to determine characteristics of differential growth and their underlying metabolic programs.

**Results:**

The cortex displayed > 5-fold higher growth than the pith during early fruit development, indicating that differential growth was established during this period. Further, when resource availability was increased through sink-removal, cortex growth was preferentially enhanced. Greatest diversity in metabolic programs between these tissues was evident during early fruit development. Higher cortex growth during early development was facilitated by increased catabolism of imported carbon (C) resources, sorbitol and sucrose, and the nitrogen (N) resource, asparagine. It was also associated with enhanced primary C metabolism, and C storage as malate and quinate. The pith metabolic program during this period involved limited allocation of C and N to growth, but greater allocation to storage, and enhanced sucrose-sucrose cycling.

**Conclusions:**

Together, these data indicate that the fruit cortex tissue displays a resource intensive metabolic program during early fruit development. This provides the C backbones, proteins, energy and osmolytes to support its higher growth.

## Background

**Fig. 1:**
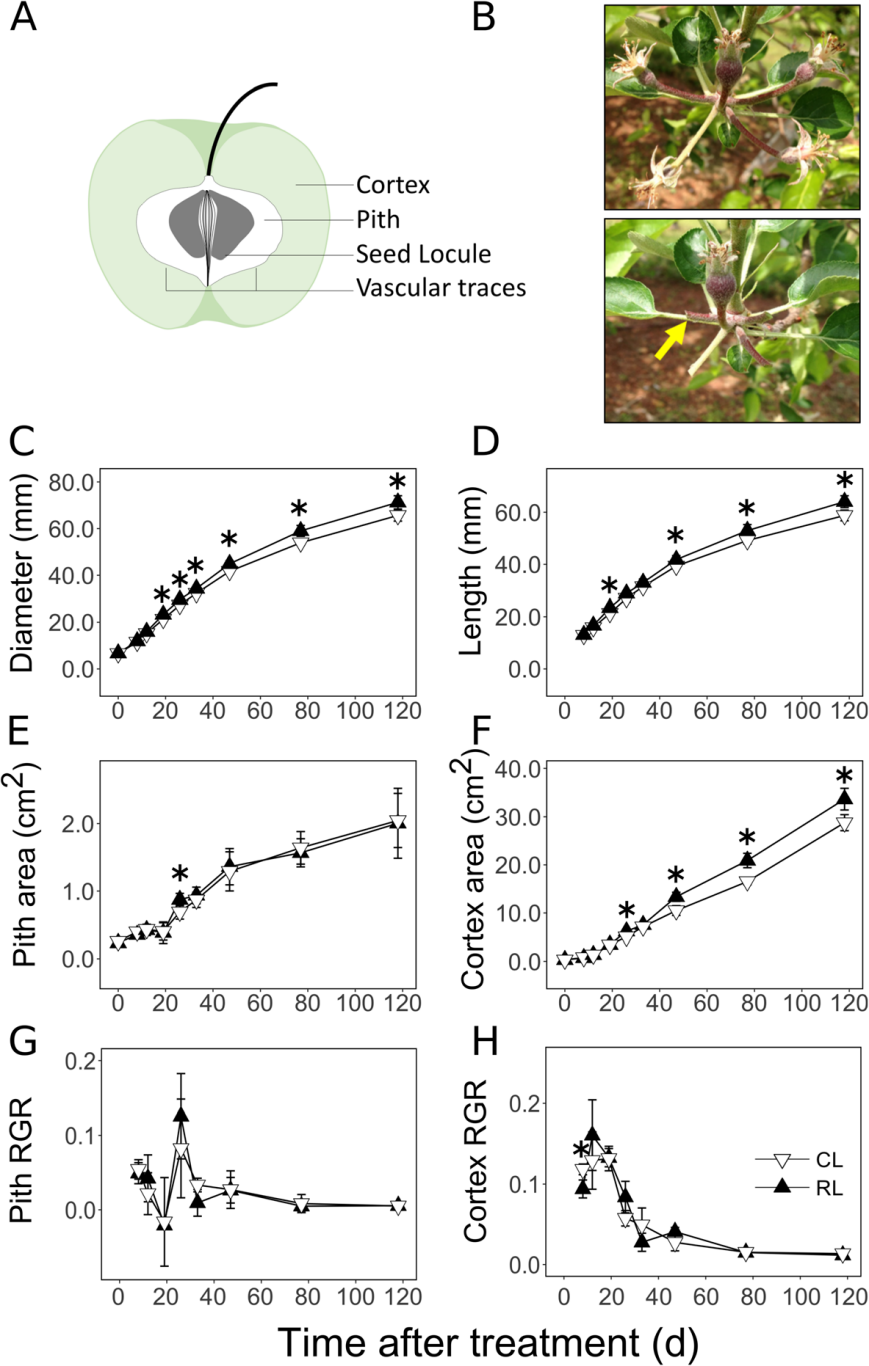
Spatial and temporal patterns of fruit growth in apple. Illustration of a longitudinal section of an apple fruit displaying the cortex, pith, seed locules and vascular traces (**a**). Apple fruit cluster before (top) and after (bottom) manual excision of fruit at the pedicel as performed for the ‘reduced fruit load’ treatment (**b**). Arrow indicates the point of fruit excision. Fruit load reduction was performed at 11 d after full bloom. Fruit diameter (**c**), length (**d**), and longitudinal sectional areas of pith (**e**) and cortex (**f**) of apple fruit are presented in relation to time after fruit load reduction treatment. Relative growth rates (RGR) of the pith and cortex tissues during fruit development and in response to fruit load reduction are displayed (**g**-**h**). Mean ± S.D. (*n* = 4) are displayed here. Asterisk indicates significant difference between the control (CL) and reduced fruit load (RL) treatments at α = 0.05

Fruits are morphologically and anatomically diverse, but in all cases are constituted by multiple tissue types of different origins. The apple fruit has two major fleshy tissues: cortex and pith (Fig. 1a). Two hypotheses, receptacular and appendicular, have been proposed to explain the origin of these tissues [1]. The receptacular hypothesis posits that the fruit fleshy tissues are of axial origin extending from the pedicel and receptacle. The appendicular hypothesis posits that the cortex which constitutes the largest portion of the fruit at maturity, is derived from accessory tissue, likely from the fused basal regions of sepals, petals and anthers [1, 2]. Further, the pith constitutes the interior region of the fruit surrounding seed locules and is thought to contain the true fruit originating from the ovary (Fig. 1a) [1, 3]. Apple fruit growth and development consist of multiple stages similar to those observed in other fruits such as tomato (*Solanum lycopersicum*) [4, 5]. Initiation of fruit growth and development occurs at fruit set following fertilization and seed set, shortly after full bloom. Fruit growth in apple is expolinear [6]. Early fruit growth involves intensive cell production (generation of new cells in a population through cell division) [7], and extends from ~ 8 until ~ 30 days after full bloom (DAFB) [8, 9]. This is followed by mid- and late-fruit growth which is linear and where the largest increase in size is achieved through extensive cell expansion by over 1000-fold [8, 10]. This period may extend until 120–180 DAFB. During the final stage of development, the fruit undergoes climacteric ripening involving changes in fruit color, reduction in firmness, reduction in starch content, increase in soluble sugars, and decrease in acidity [11, 12]. Continued growth through cell expansion can occur during this stage [13]. Cortex and pith tissues display differences in growth: while they contribute similarly to flower size at bloom, cortex constitutes > 70% of the fruit volume at maturity, indicating preferentially intensive growth of this tissue [8, 14]. The period of establishment of differential growth across these tissues during development has not been determined, although differences in tissue size are apparent by ~ 50 DAFB [8]. Mechanisms that facilitate preferential cortex growth have not yet been investigated.

Fruit growth is supported by fruit metabolism. Many fleshy fruits display limited photosynthetic capacity and are predominantly heterotrophic [15, 16]. Hence, fruit metabolism is dependent on import of carbon (C), nitrogen (N), and other resources. Carbon import is often as sucrose (Suc) in many plants [16], but predominantly as sorbitol (Sor) in apple and several other Rosaceae fruits [17]. Fruit N demands are met through remobilization of stored reserves and through new acquisition [18]. Structural and non-structural components required for fruit growth are derived from these imported resources through fruit metabolism. As the two fruit sink tissues, cortex and pith, receive inputs from the same sources, preferential cortex growth is likely supported by differential metabolic activity. Features of such spatially distinct metabolism have not been investigated previously.

Temporally dynamic metabolic programs have been identified in multiple fruits [19–22]. These studies have highlighted metabolic signatures of specific stages, and key transitions in metabolism coincident with developmental transitions. However, high resolution of temporal metabolic programs within early development, a period often involving intensive cell production, is lacking. For example, in fruits such as tomato and peach (*Prunus persica*), where metabolic programs contributing to fruit development have been investigated [16, 21, 23, 24], the period of cell production-mediated early growth was not intensively evaluated. Similarly in apple, fruit metabolite contents have been analyzed only across 1–2 stages of early development [25, 26]. Fruit metabolism during early development needs to be specific owing to the characteristic process mediating growth – rapid cell production, which requires intensive inputs for synthesis of cell wall materials, membranes and cell content, and for associated energy costs. As cell production during early development establishes a population of cells that can subsequently undergo expansion, relatively cheaply through vacuolation, growth differences established during this period may be amplified over the rest of fruit development. For example, fruit load reduction during early development enhances fruit growth by increasing cell production and this growth advantage continues through out the rest of development [27, 28]. Similarly, if the cortex metabolic program facilitates preferential growth over that of the pith during early development, this may be amplified during the rest of fruit development.

Here, we hypothesized that spatial differences in cortex and pith growth are established during early development through differential sink metabolic activities. To address this, cortex and pith tissues were analyzed at multiple stages of fruit development, particularly at high resolution during early development. Further, fruit load was altered by sink-removal, and differential growth and metabolic responses of these tissues were evaluated. To characterize the main features of metabolism, major sugar, sugar alcohol, organic acid, amino acid, and starch concentrations were quantified. Additionally, transcript abundance of multiple genes associated with metabolism of the above metabolites was determined.

## Results

### Preferential growth of fruit cortex

Fruit growth was enhanced by the fruit load reduction treatment performed at 11 DAFB [(Figs. 1b-d; 11 DAFB = 0 d after treatment (DAT)]. Fruit in the reduced fruit load (RL) treatment displayed greater diameter (9.4%; *P* < 0.01) by 19 DAT, which continued until 118 DAT. Similarly, fruit length was higher in RL fruit at 19 DAT (9.7%; *P* < 0.01) and then from 47 to 118 DAT. Pith and cortex tissues displayed differential growth during fruit development (Figs. 1e-h). They displayed similar areas at 0 DAT in RL fruit, but cortex area was larger than that of the pith in the control load (CL) fruit (*P* = 0.01). Cortex area was consistently higher than pith area under both treatments from 8 DAT. In RL fruit, pith area increased by 3.8-fold between 0 and 26 DAT, and then by 2.3-fold between 26 and 118 DAT. In the corresponding periods, cortex area increased by 19.4- and 5.4-fold, respectively (Fig. 1e and f). The relative growth rate (RGR) of the pith was generally low during fruit development (Fig. 1g). Cortex RGR was high during early development and declined from 26 DAT (Fig. 1h). Cortex RGR was higher than that of the pith during early development (0–19 DAT) by up to 6-fold. In response to fruit load reduction, cortex growth was preferentially enhanced during early stages. Cortex area in RL fruit was higher than in CL fruit by 26 DAT (23%; *P* = 0.01), and then from 47 until 118 DAT (*P* < 0.05). Cortex RGR was higher in CL fruit at 8 DAT by 1.25-fold (*P* = 0.01). Pith area was not significantly affected by fruit load reduction except transiently at 26 DAT (26%; *P* = 0.04).

### Differential metabolism across cortex and pith is most evident during early development

**Fig. 2:**
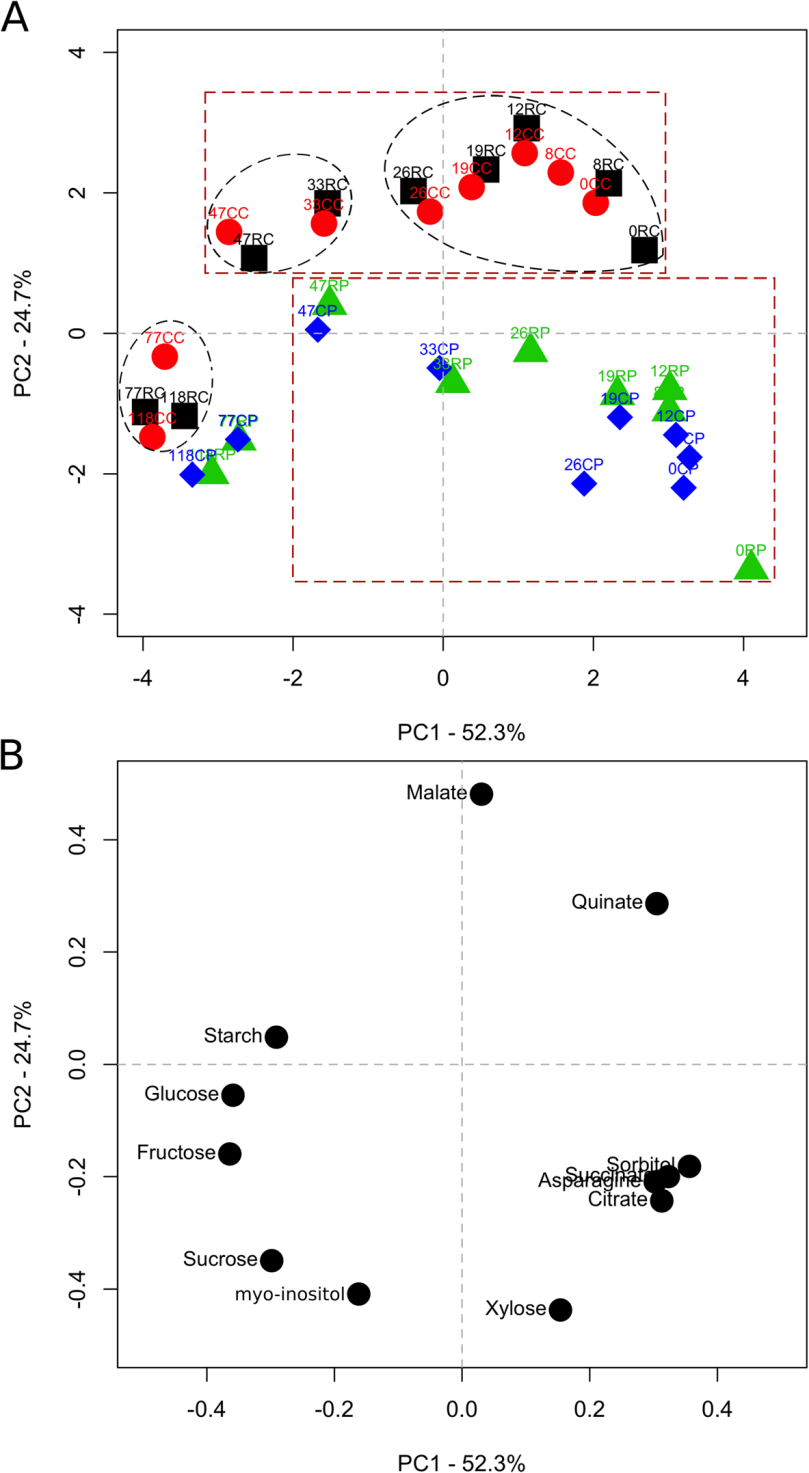
Principal components analysis (PCA) reveals spatial and temporal characteristics of apple fruit metabolism. Fruit metabolite concentration data during fruit development, across different tissue-types and in response to fruit load reduction were subjected to PCA. The first and second principal components explained 77% of variation in data and are displayed here (**a**). The ovals with dashed margins display three clusters separated based on temporal variation in fruit metabolism in the cortex. The square boxes with dashed margins display two clusters separated based on spatial variation in fruit metabolism. Fruit load reduction treatment was performed at 11 d after full bloom. The numbers above the symbols indicate days after treatment. Letters next to the numbers indicate treatment and fruit tissue type. CC: Control fruit load-Cortex (circle); CP: Control fruit load-Pith (diamond); RC: Reduced fruit load-Cortex (square); RP: Reduced fruit load-Pith (triangle). The loadings plot for principal components 1 and 2 (PC1 and PC2) are displayed (**b**). The name of the metabolite is presented next to the corresponding symbol

Metabolite concentration data were analyzed by principal components analysis (PCA). Around 77% of variance in these data was explained by two principal components, PC1 (52.3%) and PC2 (24.7%; Fig. 2a). The majority of variation was associated with temporal patterns in metabolite concentrations as the nine stages of fruit development were clearly separated along PC1. In the cortex, early stages of fruit development (0–26 DAT) were clearly separated from mid (33 and 47 DAT) and late (77 and 118 DAT) stages along PC1. A similar pattern was also evident in the pith. Hence, based on PCA, three phases of fruit development were defined: early fruit development (EFD; 0–26 DAT); mid-fruit development (MFD; 26–47 DAT); and late fruit development (LFD; 47–118 DAT). Analysis of the loading plot (Fig. 2b) indicated that fructose (Fru), glucose (Glc) and Sor contributed most to PC1 variation, and hence to the temporal patterns of variation in metabolite concentration. Cortex and pith tissues were clearly separated along PC2. Spatial separation of these data along PC2 was most prominent during EFD, and to a progressively lesser extent during later stages. Loading plot analysis indicated that malate contributed most to variation in spatial separation of metabolite data as it demonstrated the largest influence on PC2.

### Imported C is differentially catabolized in the cortex and pith during EFD

**Fig. 3:**
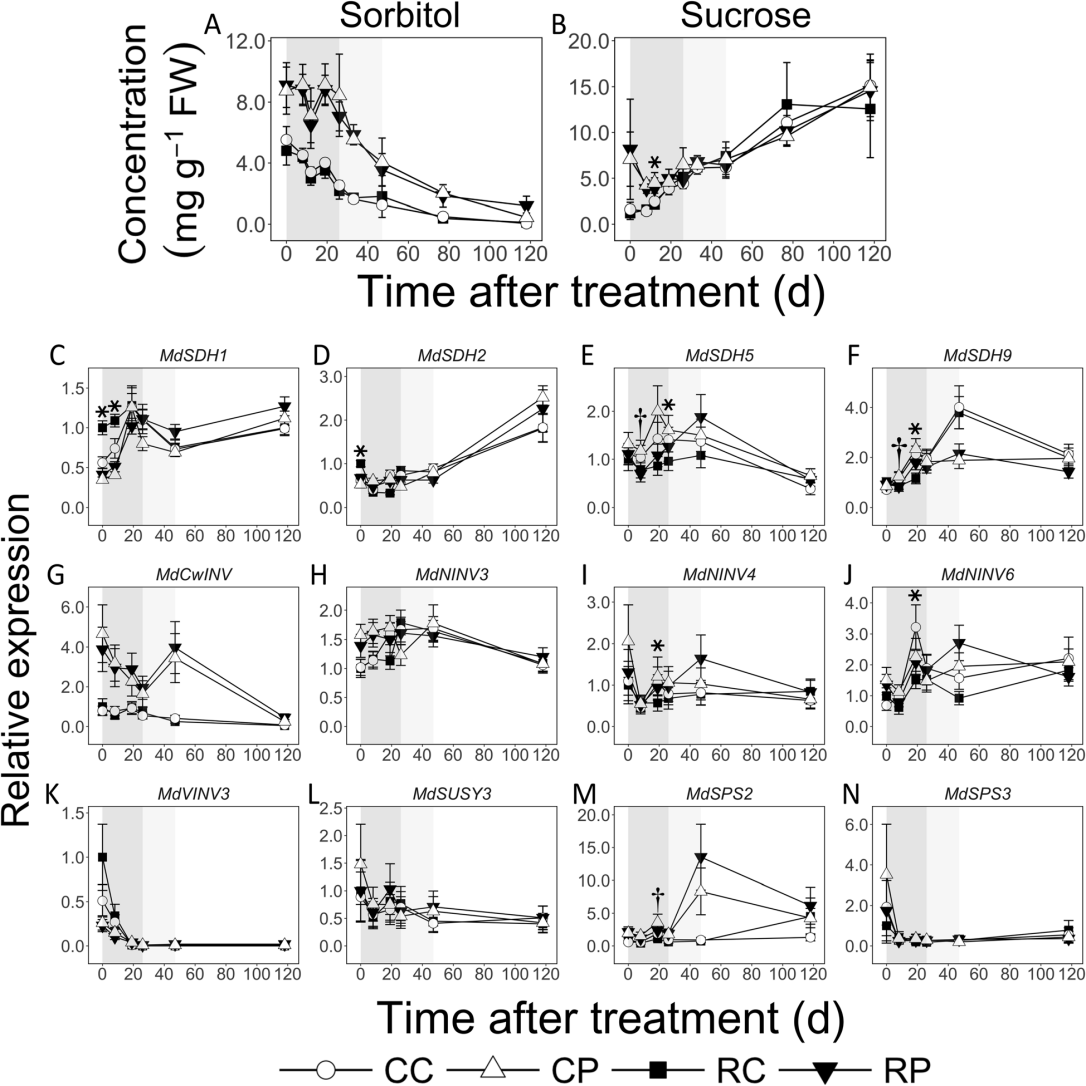
Concentrations of sorbitol (**a**) and sucrose (**b**) and transcript abundance of their metabolism-related genes (**c**-**n**) in apple fruit cortex and pith tissues in response to fruit load reduction. Means (*n* = 4) and S.D. of the mean (for metabolite concentrations) or S.E of the mean (for transcript abundance) are displayed. Transcript abundance was measured using quantitative RT-PCR. CC: Control fruit load-Cortex; CP: Control fruit load-Pith; RC: Reduced fruit load-Cortex; RP: Reduced fruit load-Pith. *SDH*: *SORBITOL DEHYDROGENASE*; *CwINV*: *CELL WALL INVERTASE*; *NINV*: *NEUTRAL INVERTASE*; *VINV*: *VACUOLAR INVERTASE*; *SUSY*: *SUCROSE SYNTHASE*; *SPS*: *SUCROSE PHOSPHATE SYNTHASE.* Transcript abundance data were normalized to that of at least two reference genes: *ACTIN* and *GAPDH* (*GLYCERALDEHYDE 3-PHOSPHATE DEHYDROGENASE*). Relative expression data are presented in reference to mean expression at 0 d after treatment in RC. The asterisk and dagger symbols indicate significant difference between control and reduced fruit load treatments in cortex and pith, respectively (α = 0.05). Treatment was performed at 11 d after full bloom. Shaded boxes in the background mark early (dark grey), mid (light grey) and late (white) fruit development

During EFD, Sor concentration declined by up to 2.2-fold in the cortex but was not altered in the pith (Fig. 3a). It was mostly higher in the pith than in the cortex by up to 3.3-fold (Eg. 26 DAT; *P* < 0.05). During MFD, it decreased in the pith and in CL fruit cortex by up to 2-fold. In the cortex, it declined further by up to 25-fold during LFD. Sucrose concentration was not significantly altered during EFD (Fig. 3b). However, it was higher in the pith than in the cortex: up to 3-fold at 8 DAT (*P* < 0.05), and 1.2-fold at 19 DAT (CL fruit; *P* = 0.01). Further, Suc concentration decreased by 13% in the cortex at 12 DAT in response to fruit load reduction (*P* = 0.04). During MFD, Suc concentration in both tissues was similar, and remained unaltered. During LFD, it increased by > 2-fold in the pith and CL fruit cortex.

Transcript abundance of four genes coding for sorbitol dehydrogenase (SDH), involved in Sor catabolism to Fru, was determined (Fig. 3c-f). *MdSDH1* and *MdSDH9* displayed an increase in transcript abundance by up to 2-fold during EFD (except in RL fruit cortex). *MdSDH1* transcript abundance was higher in the cortex during the early part of EFD by at least 2-fold (RL fruit at 0 and 8 DAT; *P* < 0.05). *MdSDH2* and *MdSDH5* transcript levels were not substantially altered during EFD and MFD. During LFD, *MdSDH2* transcript abundance increased by 2- and 3-fold in the cortex and pith, respectively. *MdSDH5* transcript abundance was 1.7-fold lower in the pith at 8 DAT, while that of *MdSDH9* was lower in the cortex by 1.8-fold at 19 DAT in response to fruit load reduction (*P* = 0.02 and 0.01, respectively).

Transcript abundance of genes coding for Suc metabolism-related enzymes was determined (Fig. 3g-n). Transcript abundance of *MdCwINV* [coding for an apoplastic invertase that catabolizes Suc to Fru and Glc] decreased by > 2-fold during EFD in the pith, but was unaltered in the cortex. *MdCwINV* transcript abundance in the pith was consistently higher than in the cortex by up to 5-fold (Eg. 8 DAT in RL fruit; *P* = 0.01). During MFD, it was higher in the pith than in the cortex by up to 16-fold (RL fruit; 47 DAT; *P* = 0.01). Among three *NINV* genes coding for neutral invertase, transcript abundance of *MdNINV3* was higher in the pith than in the cortex by 1.5-fold during the initial stages of EFD (CL fruit at 0 DAT; *P* = 0.01). Transcript abundance of *MdNINV4* and *MdNINV6* genes was reduced in response to fruit load reduction by up to 2-fold in the cortex at 19 DAT (*P* < 0.05). A vacuolar invertase gene, *MdVINV3*, displayed higher transcript abundance during EFD and remained low thereafter. Transcript accumulation of a gene coding for sucrose synthase (*MdSUSY3*), involved in Suc catabolism to Fru and UDP-Glc, was not altered in this study. Among two genes putatively coding for sucrose phosphate synthase (SPS) analyzed, *MdSPS2* displayed higher abundance in pith than in cortex tissues at multiple stages of EFD by up to 3-fold (Eg. CL fruit at 0 DAT; RL fruit at 26 DAT; *P* < 0.05), and by up to 18-fold during MFD (Eg. RL fruit, 47 DAT, *P* = 0.001). Fruit load reduction decreased its transcript accumulation in the pith by 1.5-fold at 19 DAT (*P* = 0.02).

### Cortex displays enhanced catabolism of imported N during EFD

**Fig. 4:**
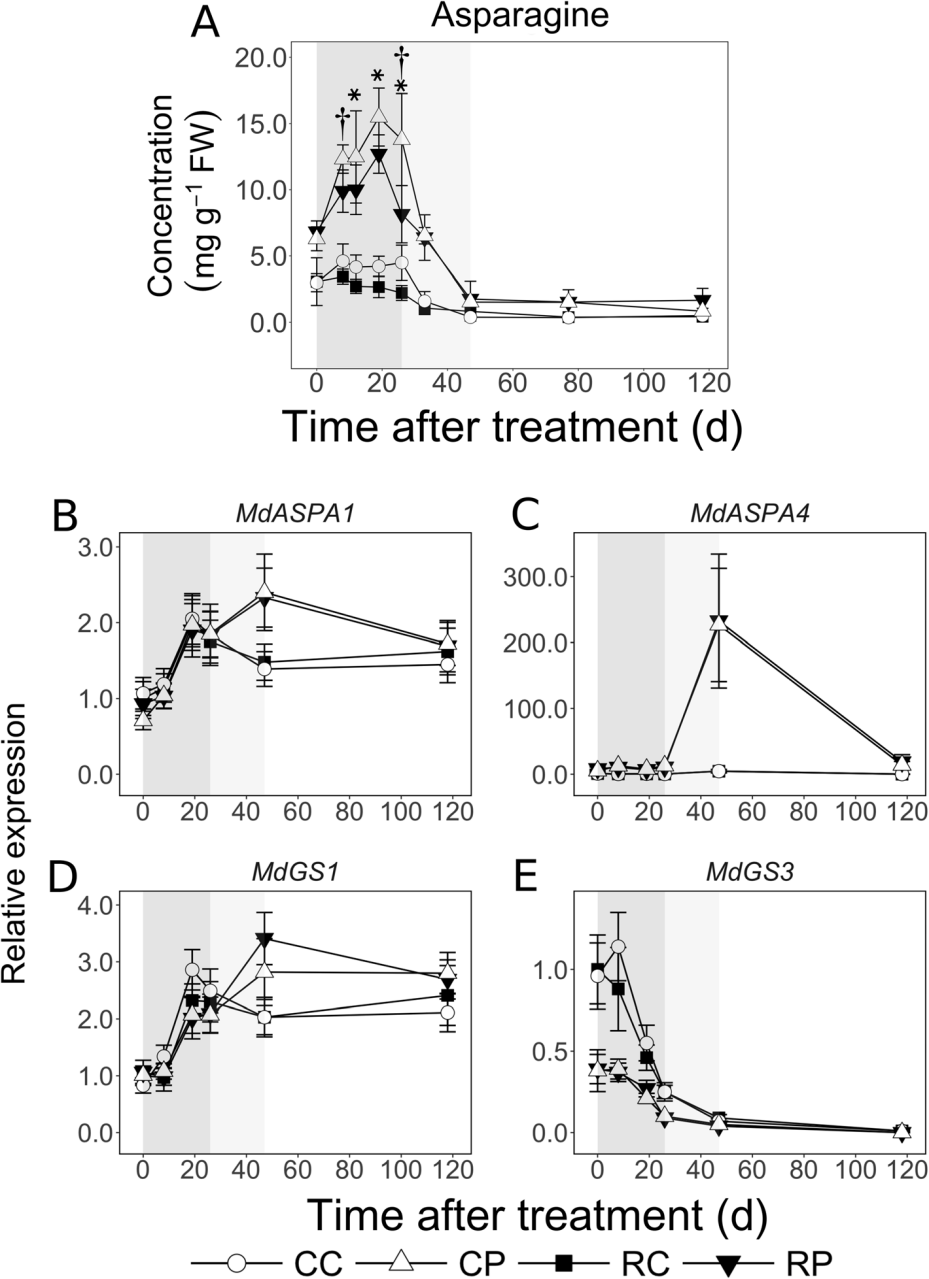
Spatiotemporal patterns of asparagine (Asn) concentration and transcript abundance of Asn metabolism-related genes in apple fruit tissues in response to fruit load reduction. Fruit load reduction was performed at 11 d after full bloom. Asparagine concentration was determined using gas chromatography and transcript abundance was determined using quantitative RT-PCR. Mean and S.D. (*n* = 4) are displayed for metabolite data. Mean and S.E. of the mean are displayed for transcript abundance data (*n* = 4). CC: Control fruit load-Cortex; CP: Control fruit load-Pith; RC: Reduced fruit load-Cortex; RP: Reduced fruit load-Pith. *ASPA*: *ASPARAGINASE*; *GS*: *GLUTAMINE SYNTHETASE*. All transcript abundance data are in reference to mean expression at 0 d after treatment in RC. Apple *ACTIN* and *GAPDH* (*GLYCERALDEHYDE 3-PHOSPHATE DEHYDROGENASE*) genes were used for normalization of transcript abundance data. Asterisk and dagger symbols indicate significant difference between control and reduced fruit load treatments in the cortex and pith, respectively (α = 0.05). Shaded regions indicate early (dark grey), mid (light grey), and late fruit development (white) periods

Asparagine (Asn) concentration in the cortex was not altered during EFD (Fig. 4a). It declined during MFD and was unaltered during LFD. In the pith, it increased by up to 2-fold during EFD, reaching peak levels by 19 DAT, declined sharply during MFD by up to 9-fold, and remained unaltered during LFD. Asparagine concentration in the pith was higher by up to 5-fold than that in the cortex through out EFD. During MFD and LFD, it was higher in the pith by up to 4-fold in CL fruit, but not in RL fruit. It decreased during EFD in response to fruit load reduction: in the pith at 8 and 26 DAT (20 and 41%, respectively; *P* < 0.05), and in the cortex at 12, 19 and 26 DAT (by 35, 37 and 51%, respectively; *P* < 0.05).

Transcript abundance of *MdASPA1*, a gene coding for asparaginase involved in Asn conversion to aspartate and ammonium, increased during EFD by up to 2-fold in both tissues (Fig. 4b). At 47 DAT, *MdASPA1* transcript abundance in the pith was > 1.5-fold higher than in the cortex (*P* = 0.02). *MdASPA4* transcript abundance was low during EFD in both tissues, but it was up to 44-fold higher in the pith than in the cortex (CL fruit, 8 DAT; *P* < 0.01; Fig. 4c). Its abundance increased dramatically in the pith by up to 22-fold during MFD concomitant with a sharp decline in Asn concentration. At 47 DAT, *MdASPA4* transcript abundance was up to 51-fold higher in the pith than in the cortex (RL fruit; *P* = 0.01). *MdGS1* (coding for a glutamine synthetase involved in assimilating ammonium) transcript abundance pattern was similar to that of *MdASPA1*, increasing during EFD by > 2-fold (by 19 DAT) and remaining unchanged during the rest of fruit development in both tissues (Fig. 4d). *MdGS3* displayed higher transcript abundance during EFD, and decreased over the rest of fruit development. Its abundance was around 2.5-fold higher in the cortex than in the pith during most of EFD (Fig. 4e; *P* < 0.05).

### Cortex and pith display differential primary C metabolism during EFD

**Fig. 5:**
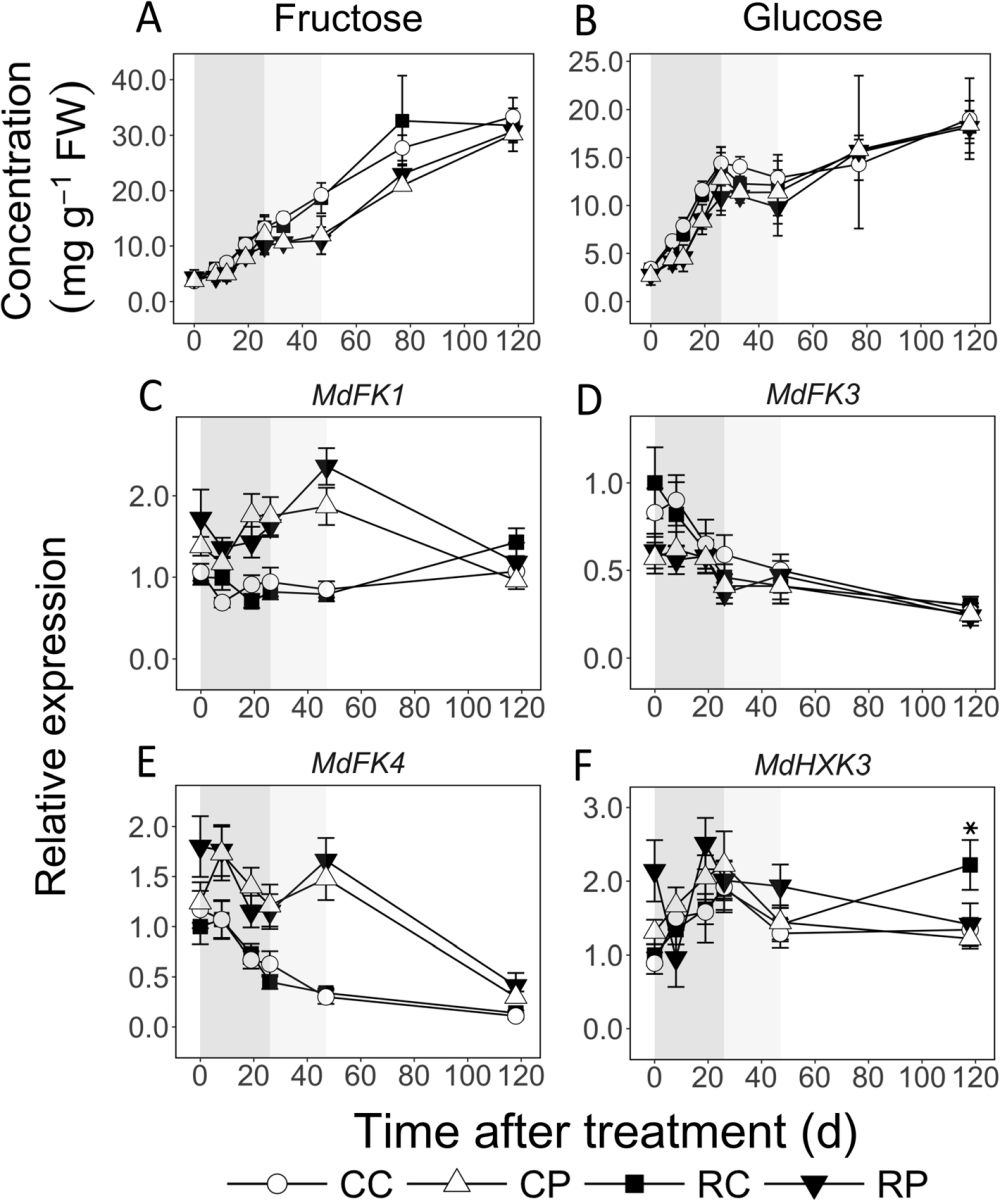
Concentrations of fructose (**a**) and glucose (**b**), and transcript abundance of their metabolism-related genes (**c**-**f**) in apple fruit tissues in response to fruit load reduction. Treatment was performed at 11 d after full bloom. Transcript abundance was measured using quantitative RT-PCR analysis. Mean and S.D. of metabolite concentration data are presented (*n* = 4). The mean and S.E of the mean (*n* = 4) of transcript abundance are displayed. CC: Control fruit load-Cortex; CP: Control fruit load-Pith; RC: Reduced fruit load-Cortex; RP: Reduced fruit load-Pith. *FK*: *FRUCTOKINASE*; *HXK*: *HEXOKINASE*. Transcript abundance is in reference to mean expression at 0 d after treatment in RC. These data were normalized to that of *ACTIN* and *GAPDH* (*GLYCERALDEHYDE 3-PHOSPHATE DEHYDROGENASE*). Asterisk and dagger symbols indicate significant difference between control and reduced fruit load treatments in the cortex and pith, respectively (α = 0.05). Shaded regions in the background indicate early (dark grey), mid (light grey), and late (white) fruit development periods

Fructose concentration increased in both tissues during EFD by up to 3.6-fold (Fig. 5a). It was higher in the cortex than in the pith at 8, 12 and 19 DAT in RL fruit and at 19 DAT in CL fruit, by up to 1.4-fold (*P* < 0.05). It continued to increase in the cortex during MFD (CL fruit), but remained unaltered in the pith. It was up to 1.7-fold higher in the cortex than in the pith. It increased during LFD in both tissues. Similarly, Glc concentration increased by over 4-fold during EFD and was higher in the cortex than in the pith (at 8, 12 and 19 DAT) by up to 1.7-fold (*P* < 0.05; Fig. 5b). During MFD, it was not altered in either tissue, and then increased during LFD by up to 1.8-fold (except in RL fruit). Transcript abundance of three *FK* genes, coding for fructokinases that catalyze phosphorylation of Fru to Fructose-6-phosphate (F6P), was analyzed (Fig. 5c-e). In the cortex, *MdFK3* and *MdFK4* transcript abundance was higher during EFD than at later stages. Transcript abundance of *MdFK1* and *MdFK4* was greater in the pith than in the cortex during most of EFD and MFD by up to 3-fold. *MdFK3* transcript abundance was higher (up to 1.5-fold) in the cortex than in the pith at 0 (RL) and 8 DAT (CL). Transcript abundance of *MdHXK3,* a gene coding for hexokinase that phosphorylates Glc to Glc-6-phosphate (G6P)*,* increased by up to 2-fold during EFD in the cortex and was not altered thereafter (Fig. 5f). Transcript abundance of these genes was mostly unaffected by fruit load reduction.

**Fig. 6:**
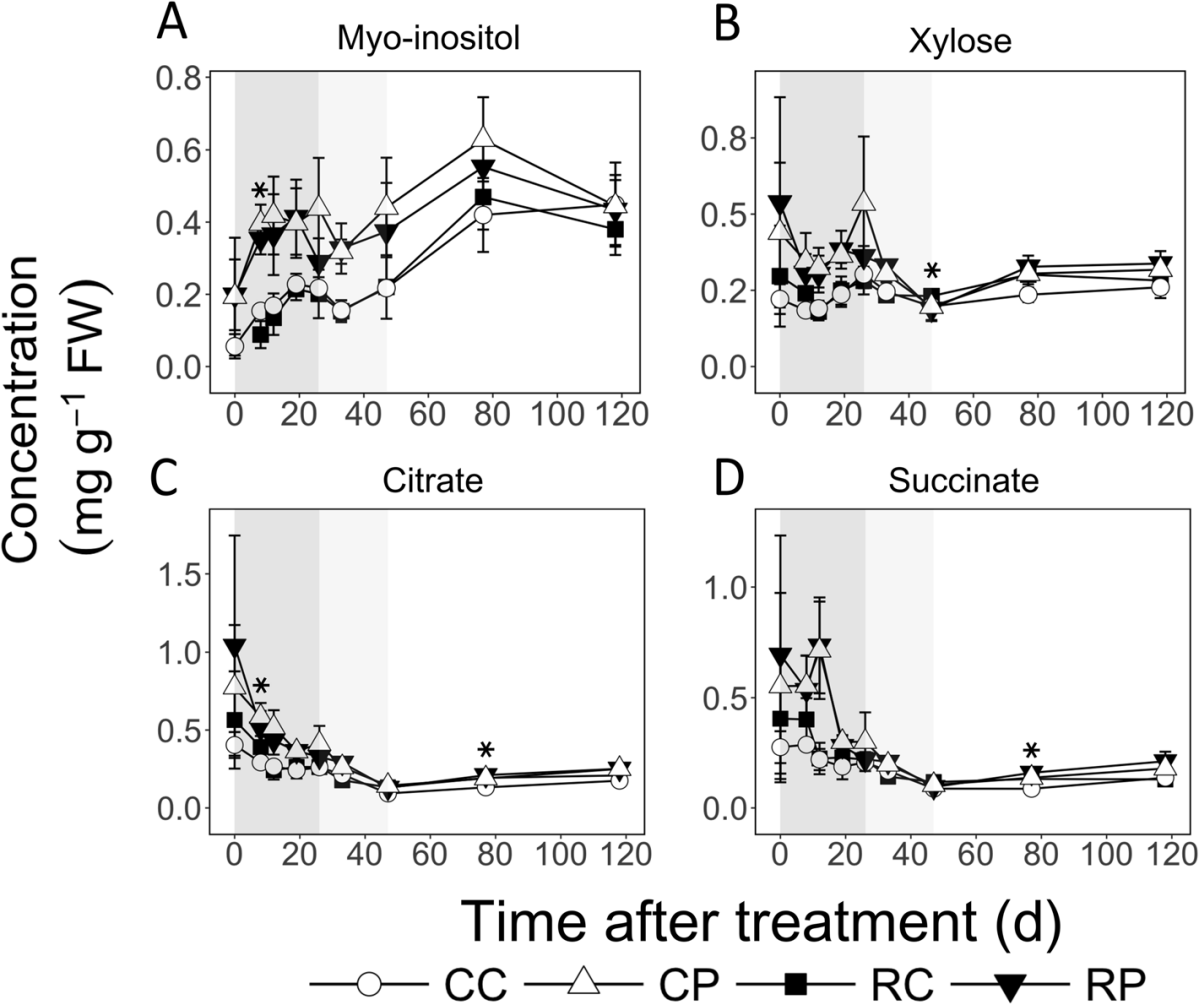
Concentrations of myo-Inositol, xylose, citrate and succinate in apple fruit tissues during fruit development and in response to fruit load reduction. The treatment was performed at 11 d after full bloom. Mean and S.D. are displayed (*n* = 4). CC: Control fruit load-Cortex; CP: Control fruit load-Pith; RC: Reduced fruit load-Cortex; RP: Reduced fruit load-Pith. Asterisks indicate significant difference between CC and RC fruit tissues (α = 0.05). Shaded regions in the background indicate early (dark grey), mid (light grey), and late fruit development (white) periods

*Myo*-inositol (Ino) concentration increased by > 3-fold in the cortex during EFD, but was not altered in the pith (Fig. 6a). It was higher in the pith than in the cortex during most of EFD by up to 3-fold, especially in RL fruit (*P* < 0.05). During MFD, it was not substantially altered but remained higher in the pith of CL fruit (2-fold; *P* < 0.05). During LFD, it increased in the CL fruit cortex by 2-fold but not significantly in the pith and the RL fruit cortex. Fruit load reduction resulted in 43% lower Ino concentration in the cortex at 8 DAT (*P* = 0.01). Xylose (Xyl) concentration was not altered during most of fruit development (Fig. 6b). Its concentration was higher in the pith during parts of EFD: at 8 DAT in CL fruit, 12 DAT in RL fruit, and 19 DAT in both (*P* < 0.05). Fruit load reduction slightly increased Xyl concentration in RL fruit cortex at 47 DAT (1.2-fold; *P* = 0.01).

Citrate concentration declined during EFD, and was higher in the pith (up to 2-fold) than in the cortex: at 8 DAT in CL fruit (*P* = 0.01), 12 DAT in RL fruit (*P* = 0.03) and 19 DAT in both (*P* < 0.05; Fig. 6c). It was not altered during the rest of fruit development. In the cortex, citrate concentration increased by around 34 and 50% due to fruit load reduction at 8 DAT and 77 DAT, respectively (*P* < 0.05). Succinate concentration was generally not altered during EFD, declined in the cortex (CL fruit) during MFD, and was not altered during LFD (Fig. 6d). During EFD, its concentration in the pith tended to be higher than in the cortex, especially in RL fruit by up to 3.3-fold (Eg. 12 DAT; *P* = 0.02). Fruit load reduction resulted in 51% higher succinate concentration in the cortex at 77 DAT (*P* = 0.01).

### Cortex and pith display differential C storage during EFD

**Fig. 7:**
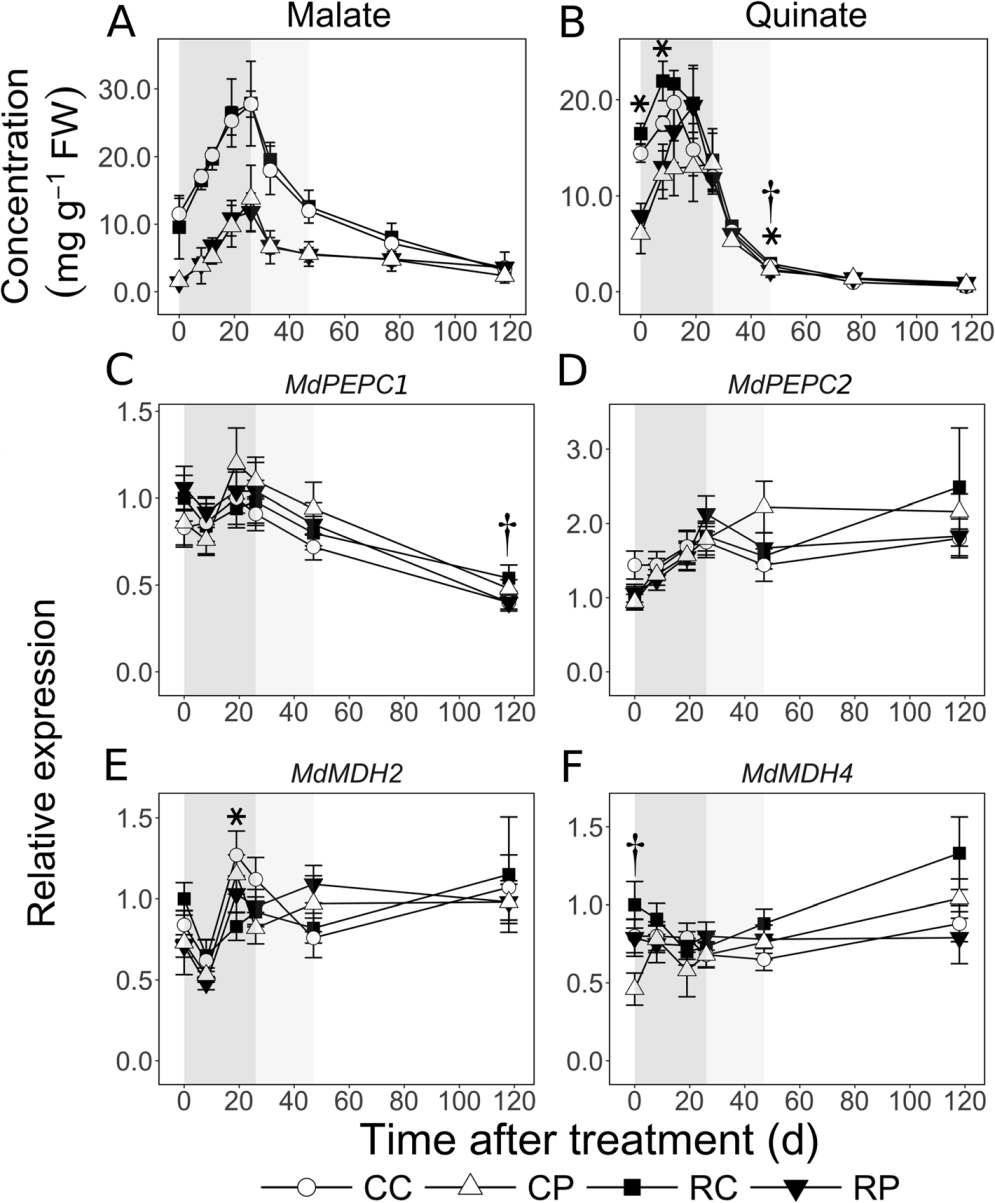
Spatiotemporal patterns of the major storage organic acids, malate (**a**) and quinate (**b**), and transcript abundance of malate metabolism-related genes (**c**-**f**) in apple fruit in response to fruit load reduction. Metabolite concentration was determined using gas chromatography and transcript abundance was measured using quantitative RT-PCR. CC: Control fruit load-Cortex; CP: Control fruit load-Pith; RC: Reduced fruit load-Cortex; RP: Reduced fruit load-Pith. Fruit load reduction was performed at 11 d after full bloom. Mean and S.D. (*n* = 4) are presented for metabolite data. The mean and S.E. of the mean (*n* = 4) are displayed for transcript abundance. Asterisk and dagger symbols indicate significant difference between control and reduced fruit load treatments in the cortex and pith, respectively (α = 0.05). *PEPC*: *PHOSPHOENOLPYRUVATE CARBOXYLASE*; *MDH*: *MALATE DEHYDROGENASE*. All expression data are presented in reference to mean expression at 0 d after treatment in RC. Transcript abundance of a target gene was normalized to that of apple *ACTIN* and *GAPDH* (*GLYCERALDEHYDE 3-PHOSPHATE DEHYDROGENASE*) genes. Shaded regions in the background indicate early (dark grey), mid (light grey), and late fruit development (white) periods

Malate and quinate were the major C storage forms during EFD. Malate concentration increased steadily by almost 3-fold in the cortex and by over 8-fold within the pith during EFD (Fig. 7a). However, it was 7-fold higher in the cortex at 0 DAT (*P* < 0.05) and consistently higher by > 2-fold during the rest of EFD. During MFD, it declined gradually by > 50% in both tissues, but remained > 2-fold higher in the cortex. During LFD, it continued to decline in the cortex, reaching similar levels in both tissues by 118 DAT. Transcript abundance of *MdPEPC1,* a gene putatively coding for phospho*enol* pyruvate (PEP) carboxylase involved in oxaloacetic acid (OAA) synthesis from PEP and HCO_3_^−^, was higher but not substantially altered during EFD, and subsequently declined by around 50% (Fig. 7c). *MdPEPC2* transcript abundance increased during EFD by up to 2-fold (except in CL fruit cortex) and was not substantially altered thereafter (Fig. 7d). Transcript abundance of *MdMDH2*, a gene coding for malate dehydrogenase that converts OAA to malate, was not substantially altered during fruit development (Fig. 7e). Fruit load reduction transiently decreased its transcript abundance by 1.5-fold in the cortex at 19 DAT (*P* = 0.02). Transcript abundance of *MdMDH4* was not altered during EFD and MFD but increased slightly during LFD in the cortex (Fig. 7f). It was higher in the pith at 0 DAT by 1.7-fold in CL fruit (*P* = 0.04). Transcript abundance of *ALUMINUM ACTIVATED MALATE TRANSPORTER* (*MdALMT9*), involved in fruit malate accumulation [29], was not substantially altered during fruit development except for an increase during EFD in CL fruit cortex (Additional file 1). It was higher in the pith than in the cortex of CL fruit at 0 DAT by 4.3-fold (*P* = 0.007). Fruit load reduction transiently reduced its expression by 1.7-fold only in the cortex at 26 DAT (*P* = 0.03).

Quinate concentration increased by up to 1.4-fold in the cortex (RL fruit) between 0 and 12 DAT and then declined (Fig. 7b). In the pith, it increased by more than 2.4-fold between 0 and 19 DAT. It was higher in the cortex than in the pith between 0 and 12 DAT by up to 2-fold (*P* < 0.05), but was similar by 19 DAT. Its concentration decreased rapidly during MFD and remained low thereafter. In CL fruit, its concentration was slightly (1.2-fold; *P* = 0.01) lower in the pith than in the cortex at 33 DAT and then higher at 77 and 118 DAT (> 1.3-fold; *P* < 0.05). Fruit load reduction resulted in higher quinate concentration in the cortex at 0 and 8 DAT by around 14 and 25% (*P* < 0.01), respectively. It also resulted in a 10 and 14% increase in concentration at 33 DAT, in the cortex and pith, respectively (*P* < 0.05).

**Fig. 8:**
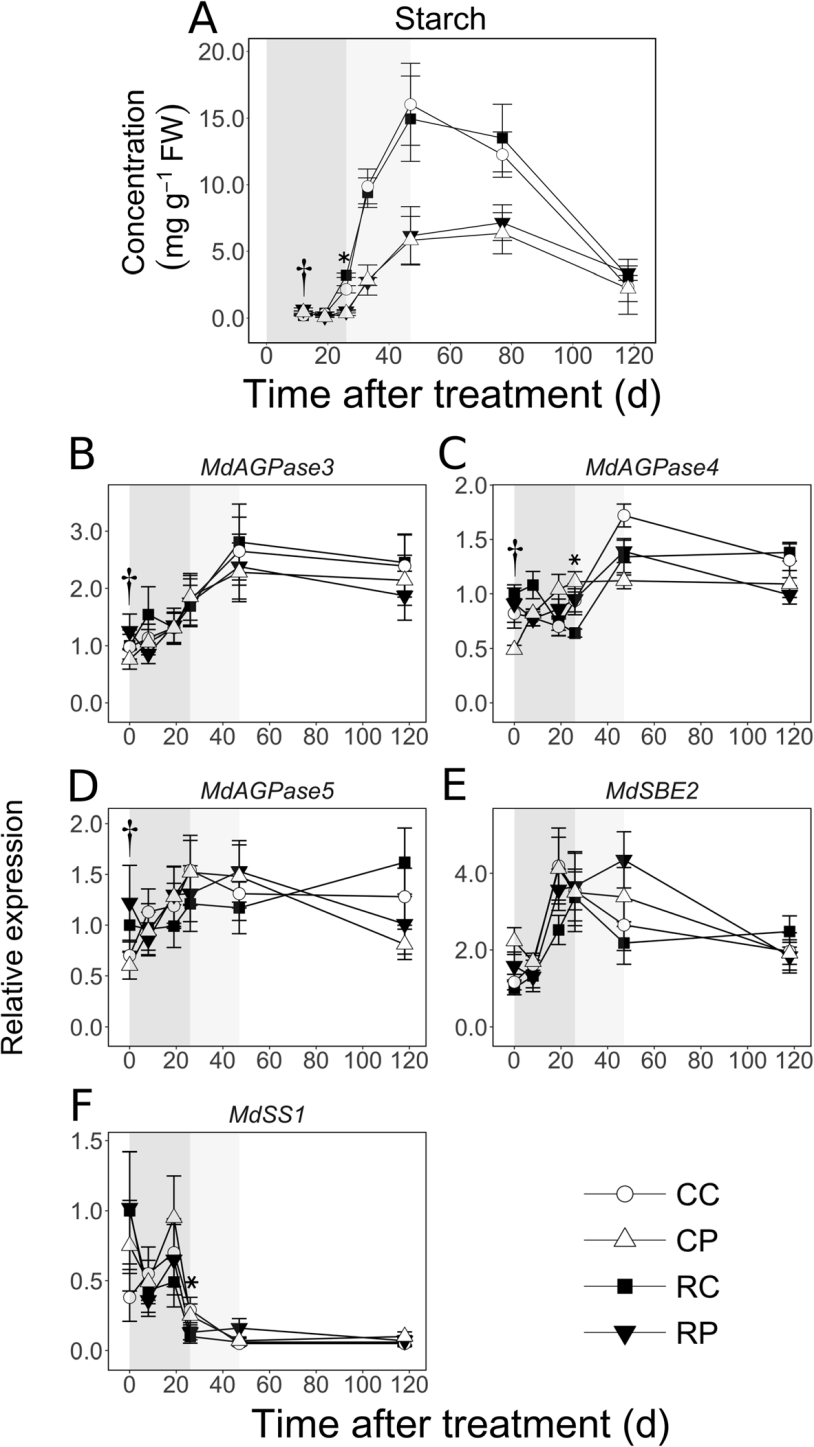
Spatiotemporal patterns of starch concentration and transcript abundance of starch metabolism related genes in apple fruit tissues in response to fruit load reduction. Fruit load reduction was performed at 11 d after full bloom. Mean and S.D. are shown for starch concentration data. The mean and S.E. of the mean (*n* = 4) are displayed for transcript abundance. CC: Control fruit load-Cortex; CP: Control fruit load-Pith; RC: Reduced fruit load-Cortex; RP: Reduced fruit load-Pith. *AGPase*: *ADP GLUCOSE PYROPHOSPHORYLASE*; *SBE*: *STARCH BRANCHING ENZYME*; *SS*: *STARCH SYNTHASE*. Asterisk and dagger symbols indicate significant difference between control and reduced fruit load treatments in the cortex and pith, respectively (α = 0.05). All expression data are in reference to mean expression at 0 d after treatment in RC tissues. Transcript abundance of a gene was normalized to that of apple *ACTIN* and *GAPDH* (*GLYCERALDEHYDE 3-PHOSPHATE DEHYDROGENASE*) genes. Shaded regions indicate early (dark grey), mid (light grey), and late fruit development (white) periods

Starch concentration could not be quantified at 0 and 8 DAT due to limited tissue availability. Its concentration was low (< 0.7 mg g^− 1^) in both tissues at 12 and 19 DAT (Fig. 8a), but up to 3.5-fold higher in the pith at 12 DAT (*P* < 0.05). Fruit load reduction resulted in higher starch concentration in the pith by 1.4-fold at 12 DAT (*P* = 0.02), and in the cortex by 1.5-fold at 26 DAT (*P* < 0.001). Starch concentration increased greatly during MFD: by 4- to 7-fold in the cortex, and by up to 16-fold in the pith. However, it was still lower in the pith than in the cortex by 2- to 3-fold during this period. During LFD, it declined in both tissues, primarily between 77 and 118 DAT.

Transcript abundance of three genes coding for ADP-glucose pyrophosphorylase (*MdAGPase3*, *MdAGPase4* and *MdAGPase5*), an enzyme catalyzing synthesis of ADP-Glc from Glc-1-phosphate (G1P), was determined (Fig. 8b-d). *MdAGPase3* transcript abundance was increased by up to 2-fold between 19 and 47 DAT (except in RL fruit pith). *MdAGPase4* transcript abundance increased by > 2-fold in the cortex during MFD, and was higher in the CL fruit cortex than in the pith by 1.5-fold (*P* = 0.02). *MdAGPase5* transcript abundance was not substantially altered during fruit development. Transcript abundance of a gene coding for a starch branching enzyme, *MdSBE2*, increased during EFD, in cortex and in RL fruit pith by over 3-fold and 2-fold, respectively (Fig. 8e). It was higher in the pith by up to 1.9-fold at 0 DAT (*P* < 0.05). Transcript accumulation of *MdSS1,* a gene coding for starch synthase, was higher during EFD, declined between 19 and 26 DAT (except in CL fruit cortex), and remained low thereafter (Fig. 8f). Fruit load reduction resulted in 65% lower *MdSS1* transcript abundance in the cortex at 26 DAT (*P* = 0.01).

## Discussion

### Fruit cortex displays preferential growth during EFD

Spatial differences in growth were clearly established during EFD (Fig. 1). Cortex growth during EFD was > 5-fold higher than that in the pith. Majority of cortex growth during EFD is associated with rapid cell production [9, 10, 28]. Hence, higher cortex growth was likely achieved through greater cell production than that in the pith. Further, fruit load reduction enhanced growth primarily in the cortex, and this was apparent by the end of EFD (Fig. 1). Fruit load reduction enhances early fruit growth by increasing cell production [9, 10, 28]. Hence, additional resources translocated into the fruit due to sink-removal [30, 31] are allocated primarily to enhancing cell production allowing for greater cortex growth. Together these data indicate that cortex is established as the dominant sink tissue during EFD through enhanced cell production.

### Differential cortex sink activity supports its preferential growth during EFD

Fruit metabolism during EFD was clearly separable from that at later stages (Fig. 2), indicating that cell production-mediated growth requires a specific metabolic program. Similarly, EFD was metabolically separable from later stages of development in peach [21]. The greatest divergence between cortex and pith tissues in metabolite concentrations was noted during EFD (Fig. 2), indicating that their differential growth was facilitated by differential sink metabolic activities during this period. Key characteristics of such differential sink metabolism during EFD are discussed below.

**Fig. 9:**
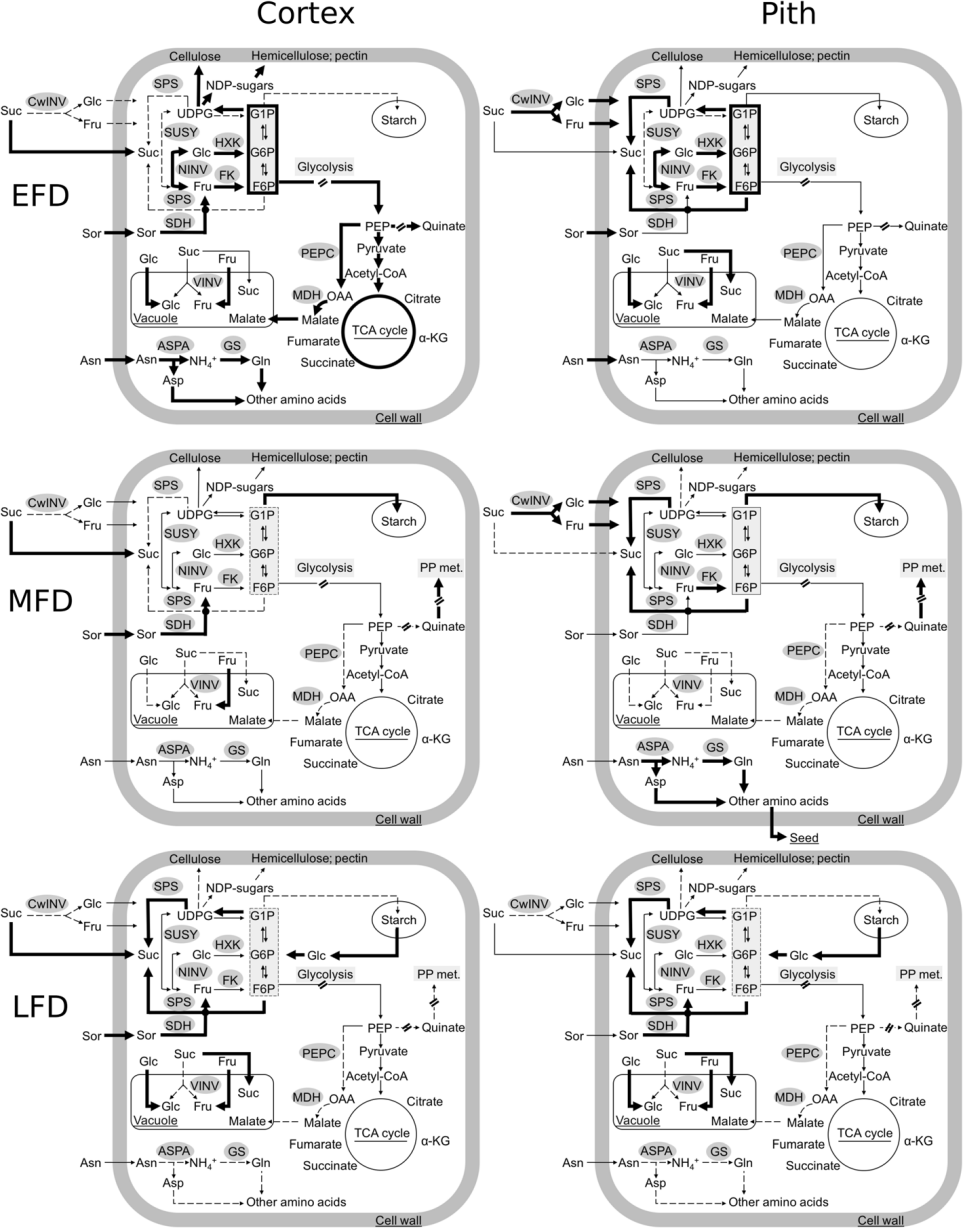
Spatiotemporal metabolism in the apple fruit. Fruit metabolite and transcript abundance data from fruit cortex and pith tissues during different stages of development were used to develop a representative model of fruit cell metabolism. Space outside of the cell boundary is represented as the apoplastic space (includes cell wall). EFD: early fruit development; MFD: mid fruit development; LFD: late fruit development. Enzyme nomenclature: CwINV: cell wall invertase; NINV: neutral invertase; VINV: vacuolar invertase; SDH: sorbitol dehydrogenase; SUSY: sucrose synthase; SPS: sucrose phosphate synthase; FK: fructokinase; HXK: hexokinase; ASPA: asparaginase; GS: glutamine synthetase; MDH: malate dehydrogenase; PEPC: phospho*enol*pyruvate carboxylase. PP metabolism indicates the phenylpropanoid metabolic pathway. Metabolite nomenclature: Sor: sorbitol; Suc: sucrose; Fru: fructose; Glc: glucose; F6P: fructose-6-phosphate; G6P: glucose-6-phosphate; G1P: glucose-1-phosphate; UDPG: uridine diphosphate-glucose; NDP-sugars: nucleoside diphosphate-sugars; PEP: phospho*enol*pyruvate; OAA: oxaloacetic acid; Asn: asparagine; Gln: glutamine; Asp; aspartate; α-KG: α-keto glutarate. Bold arrows indicate higher flux; narrow arrows indicate medium flux and dashed lines indicate reduced flux

Higher cortex growth was associated with greater catabolism of imported C resources, likely to provide C backbones and energy required to support intensive cell production (Fig. 9). Rapid cortex growth was associated with greater Sor catabolism as its concentration declined in this tissue by over 2-fold during EFD, and was over 2-fold lower than that in the pith (Fig. 3). Sorbitol metabolism is chiefly mediated by SDH, which displays high activity during EFD and contributes to fruit sink strength [32–34]. Transcript abundance of *MdSDH1* and *MdSDH9* increased in the cortex during EFD in a pattern complimentary to the decline in Sor concentration, and *MdSDH1* abundance was higher in the cortex than in the pith, implicating their gene products in its metabolism. *MdSDH1* and *MdSDH9* transcript abundance also increased in the pith during late stages of EFD, where Sor concentration was not substantially altered. Potentially, post-translation modifications regulate SDH activity [35] in the pith during this period. Sucrose concentration was also lower in the cortex during initial stages of EFD and declined transiently in response to fruit load reduction. These data suggest that Suc catabolism also supported the high C demand for cell-production mediated growth. In contrast, lower growth in the pith during EFD was associated with substantial allocation of imported C to storage. Sorbitol concentration was higher in the pith, suggesting greater storage. There was evidence of greater Suc-Suc cycle activity resulting in higher Suc levels in pith cells. Transcript abundance of *MdCwINV* was up to 5-fold higher in the pith during EFD (Fig. 3g), suggesting greater apoplastic Suc catabolism and differential entry of Suc-derived C into pith cells: as Glc and Fru (Fig. 9). Transcript abundance of *MdFK1*, *MdFK4* and *MdSPS2* were coordinately higher by up to 3-fold during EFD, and consistently Suc concentration was up to 3-fold higher (Figs. 3 and 5), suggesting enhanced conversion of the hexoses to Suc. Further, greater C storage as starch was noted in the pith during a part of EFD (12 DAT). Together, these data suggest that the pith metabolic program during EFD facilitated allocation of a greater proportion of imported C to storage (Fig. 9).

Differential sink activities across cortex and pith tissues also involved differential N metabolism during EFD. Concentration of the primary imported N source in apple, Asn [36, 37], was > 3-fold lower in the cortex than in the pith, and it declined further in response to fruit load reduction (Fig. 4). Cell production-mediated growth requires high N inputs [38], especially for protein synthesis. Consistently, increasing N supply enhances cell production and fruit growth [39]. Higher N demands during EFD in the cortex, and in response to fruit load reduction, may be met by intensive Asn catabolism. Consistent with this, transcript abundance of *MdASPA1* and *MdGS1* increased in a coordinated manner. Further, *MdGS3* transcript abundance was specifically higher in the cortex by > 2-fold, indicating that downstream N metabolism was also enhanced in this tissue. Together these data indicate that during EFD, metabolism of imported N is elevated in the cortex to support intensive demands of early growth, while imported N accumulates in the pith (Fig. 9).

Differential sink metabolic activity in the cortex also involved elevated primary C metabolism to meet structural and energy demands of cell production-mediated growth during EFD. Fructose in apple fruit cells is derived from Sor and Suc catabolism. It can subsequently accumulate in the vacuole or be phosphorylated to F6P by FK for downstream metabolism. In the cortex, multiple lines of evidence suggest that a higher proportion of Fru was allocated to downstream metabolism during EFD. In spite of higher Sor catabolism in the cortex, Fru accumulation was not proportionately higher in this tissue than that in the pith. In the cortex, transcript abundance of two *FK* genes was higher during initial stages of EFD and declined at later stages (Fig. 5), suggesting higher fructokinase activity during EFD, consistent with previous reports [40–42]. Fructose-6-phosphate can contribute to Suc synthesis through SPS activity [43]. However, as the cortex Suc concentration was relatively lower than that in the pith during initial stages of EFD, a higher proportion of F6P was likely allocated to alternative metabolism via the hexose-phosphate pool (F6P, G1P and G6P). Glucose also contributes to the hexose-phosphate pool following its phosphorylation to G6P by hexokinase [43]. Consistently, transcript abundance of *MdHXK3* increased in the cortex during EFD, suggesting substantial conversion of Glc to G6P. Similarly, the cell division phase of tomato fruit development was associated with higher activity of enzymes associated with hexose phosphorylation [44]. Hexose-phosphates have multiple fates with a major one as allocation to respiration. Concentration of the TCA cycle metabolites, citrate and succinate, was lower during EFD in the cortex, suggesting higher extent of glycolysis and TCA in this tissue, and consistent with previously reported high respiration rates during this period [15, 26]. Hexose-phosphates can also be allocated to cell wall synthesis through generation of nucleotide-sugars [45]. As cell production-mediated growth was higher in the cortex, a higher proportion of the hexose-phosphate pool may be allocated to new cell wall synthesis in this tissue (Fig. 9). Hexose-phosphates may also be allocated to synthesis of Ino which contributes to cell structural component synthesis, as a precursor for synthesis of cell walls (pectin) and membrane components [46]. Myo-Inositol concentration was 3-fold lower in the cortex and decreased further under fruit load reduction (Fig. 6), consistent with greater allocation of Ino towards supporting structural component synthesis associated with enhanced cell production. Together, these data indicate that higher extent of primary C metabolism during EFD supports structural and energy demands of rapid cell production-mediated cortex growth (Fig. 9).

High respiration during EFD results in greater CO_2_ release which can be re-fixed (as HCO_3_^−^) by PEPC using PEP to ultimately generate malate, which is stored as a C reserve in the vacuole [47]. Consistently, higher growth (and associated respiration [26]) in the cortex during EFD may allow for greater re-fixation of the released C, resulting in higher malate concentration in this tissue (Fig. 9). PEP along with erythrose-4-phosphate (E4P), also serves as a substrate for synthesis of dehydroquinate, a precursor of quinate and shikimate [48]. Quinate concentration increased during EFD, was higher in the cortex, and was enhanced in response to fruit load reduction (Fig. 7) indicating substantial C allocation via PEP to quinate. PEP also serves as a substrate for pyruvate synthesis which allows for C entry into the TCA cycle. In the cortex, higher malate and quinate synthesis via PEP may reflect a metabolic program that allows for fine-tuning C allocation to respiration to meet the dynamic energy demands of growth, for C storage during EFD to meet energy and C skeleton demands at later stages, and for facilitating subsequent cell expansion (as osmolytes).

### Differential metabolism continues during MFD and LFD

Growth during MFD is primarily mediated by post-mitotic cell expansion [9, 10, 28] and likely facilitated by metabolite accumulation in the vacuole. Differential metabolic activities across fruit tissues apparent during this period were largely related to C storage (Fig. 9). A characteristic feature of MFD was the rapid increase in starch concentration which occurred at a higher rate in the cortex than that in the pith (Fig. 8). This was likely supported by higher C partitioning to the cortex owing to its greater sink size. Cell production, a major resource sink, ceases prior to MFD and growth is mediated largely by post-mitotic cell expansion [10, 28, 49]. Consequently, a substantial proportion of imported C may be re-allocated from multiple metabolic routes to starch biosynthesis (Fig. 9) [30, 50]. Increase in starch concentration coincided with a sharp reduction in malate and quinate concentrations suggesting that C was re-allocated from storage as organic acids. In both tissues, increase in Glc concentration observed during EFD was halted at the onset of MFD, and its tissue content increased at a lower rate during this period than that during EFD (Additional file 3). Similarly, a temporary decrease in Glc concentration during the starch accumulation period was reported previously [50]. These data are consistent with diversion of C from Glc accumulation to starch synthesis. In the cortex, Fru was allocated primarily to storage consistent with previous reports [51] as Sor concentration decreased slightly while Fru concentration continued to increase in a correlated manner (Additional file 2). However, in the pith, Fru concentration did not increase even though Sor concentration declined by over 2-fold, and apoplastic Suc catabolism was higher (transcript abundance of *CwINV* was > 10-fold higher in the pith; Fig. 3). Further, transcript abundance of *MdFK1* and *MdFK4* was higher in the pith (Fig. 5), suggesting enhanced F6P synthesis. Also, *MdSPS2* transcript abundance was higher in the pith by over 18-fold (Fig. 3) suggesting enhanced Suc re-synthesis. However, as Suc concentration and content (Additional file 3) did not increase proportionately, it is likely that newly synthesized Suc was rapidly diverted to starch synthesis in the pith, potentially through SUSY and UDP-Glc pyrophosphorylase activities [30].

The pith displayed increased catabolism of imported N during MFD. Asparagine concentration declined sharply in the pith at a greater rate than that in the cortex (Fig. 4). This was associated with a steep increase in *MdASPA4* transcript abundance, and relatively higher abundance of *MdASPA1* and *MdGS1* transcripts, suggesting transcriptionally regulated enhanced Asn catabolism and subsequent N metabolism in the pith. Potentially, this provides amino acids to support N requirements for adjacent seed development (Fig. 9).

While differential sink activities were still evident at LFD, they were substantially less prominent than during earlier stages. LFD is associated with growth mediated by post-mitotic cell expansion [10, 49], and ripening. Continued accumulation of Fru, Glc and Suc during LFD allows for maintenance of vacuolar osmolytes needed for continued cell expansion-mediated growth during this period. Fructose concentration continued to increase, supported by Sor catabolism likely mediated by an increase in *MdSDH2* transcript abundance, and was higher in the cortex (CL fruit). Starch concentration decreased dramatically during LFD, especially in the cortex. This likely contributes to resumption of increases in Glc and Suc concentrations, and partly meets metabolic requirements of a respiratory climacteric [30].

## Conclusion

Differential growth across two major apple fruit fleshy tissues, cortex and pith, is primarily established during EFD, and is supported by their differential sink metabolic activities. Higher catabolism of imported C and N resources, greater primary C metabolism, and higher C storage as organic acids are the hallmarks of differential cortex metabolic activity. Together, these features provide C backbones, proteins, energy and osmolytes required for greater cell production and establishment of cortex as the dominant sink during EFD, a growth advantage that continues through out the rest of fruit development. Pith growth is sink-limited during EFD and its metabolic activity primarily involves C and N allocation to storage, and enhanced Suc-Suc cycling. Knowledge of these contrasting metabolic features can aid in efforts to optimize organ growth. Further, physical features such as development of transport structures including vascular tissues may differ across these tissues [52]. Such characteristics may also contribute to differential growth and need further investigation. Together, differential growth of these adjacent fleshy tissues presents an excellent model system to explore molecular regulation of fruit growth, metabolism and their inter-dependence.

## Methods

### Plant material

Mature apple trees of the widely cultivated genotype, ‘Golden Delicious Smoothee’, on M.7a rootstock at the Mountain Research and Education Center, University of Georgia, Blairsville, GA, were used in this study. These trees were planted in 2001 and were identified as such by the research staff. The trees were maintained following regional commercial production practices by the research staff. In 2015, four trees were randomly selected and subjected to the fruit load reduction treatment (RL) while another four trees were untreated and used as controls (CL). Each tree served as an experimental unit. For the RL treatment, all fruit except the central (king) fruit within a fruit cluster were manually removed at 11 DAFB by excising the pedicel ~ 1 cm below the fruit using scissors (Fig. 1b). Previous studies indicated that fruit removal at this stage enhances fruit growth by stimulating cell production [10, 28]. Hence, at the initiation of this experiment, fruit load in the CL treatment was potentially 5-fold higher than that in the RL treatment. However, owing to several cycles of physiological fruit drop in apple beginning around 10–15 DAFB, this ratio changes over fruit development. Fruit removal studies performed previously with this genotype at the same developmental stage and location resulted in ~ 3-fold difference in fruit load between CL and RL treatments at harvest [28]. Fruit of this genotype typically reach harvest maturity around 140–150 DAFB [28, 53]. Chemical thinning agents were not applied during this study to avoid interference with fruit growth. Fruit diameter and length were measured on 10 tagged king fruit from each tree at 0 (11 DAFB), 8, 12, 19, 26, 33, 47, 77, and 118 DAT (Additional file 4). At each stage, four king fruit from each tree were sampled and longitudinally cut in half. One half was fixed in CRAF III (Chromic acid: acetic acid: formalin) fixative for morphometric analysis, and the other half was used for determining metabolite and transcript abundance. For latter use, pith and cortex tissues were separated using biopsy punches (1–10 mm), immediately frozen in liquid nitrogen, and stored at − 80 °C. Both tissues were uniformly sampled during all stages of fruit development to avoid potential zone effects.

### Measurement of pith and cortex growth

Images of the longitudinal fruit profile were obtained using a flatbed scanner (V600, Epson). ImageJ (National Institutes of Health, USA) was used to outline and measure locule, core (marked by sepal and petal vascular traces) and total fruit sectional areas (Fig. 1a). Area of the core was subtracted from that of the fruit to obtain cortex area. Area of locule was subtracted from that of the core to obtain pith area. Tissue RGR was determined from the area measurements as: *[Ln(A*_*2*_*) – Ln(A*_*1*_*)] / [T*_*2*_ *- T*_*1*_*]*, where *A*_*2*_ and *A*_*1*_ are areas at two consecutive time points, *T*_*2*_ and *T*_*1*_, respectively.

### Metabolite measurement using gas chromatography (GC)

Metabolites were extracted and analyzed according to [54] with some modifications. Fruit tissues were ground in liquid nitrogen. Around 50–100 mg of ground tissue was extracted in 1.2 mL of 80% methanol containing phenyl β-D-glucoside as an internal standard. After centrifugation for 40 min at 14,000 *g* and 4 °C, 100 μL of the supernatant was transferred to a 300 μL glass insert in a 2 mL GC vial. The solvent was evaporated under a stream of nitrogen. Metabolites were first converted to their oxime derivatives by adding 25 μL hydroxylamine and heating to 50 °C for 30 min, and then converted to their tri-methyl silyl (TMS) derivatives by adding 50 μL of BSTFA (Bis (trimethylsilyl)trifluoroacetamide) and heating to 50 °C for 30 min. One μL of this mixture was injected and analyzed on a gas chromatograph (GC-2014; Shimadzu, Japan) equipped with an HP-5 capillary column (Agilent Technologies Inc., USA) and a flame ionization detector. Helium was used as the carrier gas. The oven temperature program was: 1 min at 150 °C, 4 °C/min ramp to 190 °C, 0.5 min at 190 °C, 1.5 °C/min ramp to 210 °C, 0.5 min at 210 °C, 10 °C/min ramp to 260 °C, 10 min at 260 °C. Standard solutions were prepared for all metabolites, and derivatized as described above. Standard curves were generated and used for metabolite quantification. Metabolites analyzed using GC in this study were: Sor, Suc, Glc, Fru, Xyl, Ino, malate, quinate, citrate, succinate, and Asn.

### Starch quantification

Starch concentration was determined as mg Glc equivalents g^− 1^ fresh weight following [55]. Around 50–100 mg of ground tissue was extracted three times in 80% ethanol at 80 °C for 10 min. The pellet was retained and digested with 35 units of amyloglucosidase at pH 4.8 and at 55 °C for 36 h. Glucose concentration was measured using an enzymatic assay in which hexokinase and Glc-6-phosphate (G6P) dehydrogenase were used. NADH generated during the conversion of Glc to 6-phosphogluconate was monitored spectrophotometrically at 340 nm. A standard curve of Glc was used to determine the equivalents.

### RNA extraction, cDNA synthesis, and qPCR

Total RNA was extracted using the CTAB extraction buffer method described previously [56]. Synthesis of cDNA was performed using 1 μg of total RNA. ImProm II reverse transcriptase (Promega, USA) was used for reverse transcription in a volume of 20 μL. The cDNA was diluted 6-fold, and 1 μL of diluted cDNA was used for quantitative RT-PCR following the method described previously [28], with the exception of using PowerUp SYBR green master mix (ThermoFisher, USA). A Stratagene Mx3005P (Agilent Technologies, USA) quantitative real-time PCR instrument was used. Melt-curve analyses were performed at the end of PCR amplification to determine primer specificity. Control reactions without template were included. Two reference genes were used for normalization of target gene expression, *MdACTIN* and *MdGAPDH*. In case of *MdSDH1*, *MdSDH2*, *MdNINV3* and *MdSUSY3*, an additional reference gene, *MdCACS*, was used. Selection of genes for analysis was based on [40], and on highest abundance within a gene family based on RNA-Seq data (Jing and others, *In preparation*). List of genes and the primer sequences for qRT-PCR are presented in Additional file 5. Efficiencies of qPCR reactions were determined using LinRegPCR [57]. Relative quantity (RQ) values were determined following efficiency correction and normalized using the geometric mean of RQs of reference genes to generate normalized RQs (NRQs). Data analysis were performed on NRQ values after log_2_ transformation. Standard errors were determined as described in [58]. Expression of all genes are presented as fold change in relation to mean transcript abundance of target gene in RL fruit cortex at 0 DAT. Only differences in transcript abundance statistically significant and > 1.5-fold different are discussed.

### Statistical analyses

Statistical analyses and graph preparation were performed using RStudio (Version 1.0.143) and Inkscape (Version 0.92.3). Temporal changes in fruit diameter, length, cortex area and pith area within a treatment were analyzed using analysis of variance (ANOVA; α = 0.05) followed by mean separation using Tukey’s honestly significant difference (HSD; α = 0.05). These data were compared between CL and RL fruit using *Student’s t-tests* (α = 0.05) at each stage. Cortex and pith areas were compared within a stage and fruit load treatment using paired *t-tests* (α = 0.05). Metabolite concentration and transcript abundance differences between tissues were analyzed within a given stage and fruit load treatment using paired *t–tests* (α = 0.05), and between CL and RL treatments at a given stage and tissue-type using *Student’s t-tests* (α = 0.05). Temporal changes in the above data within a fruit load treatment and tissue-type were analyzed using ANOVA (α = 0.05) followed by Tukey’s HSD (α = 0.05). Statistical significance of these data are presented in Additional file 6. Principal components analysis (PCA) was performed using metabolite concentration data from nine stages, two tissue types, and from two fruit load treatments. These data were used to identify the major determinants of variation in metabolite concentrations with the *prcomp* function in RStudio. The first two principal components which explained most of the variation and their loadings plot are displayed using the plot function in RStudio.

## Supplementary information

**Additional file 1. **Spatiotemporal patterns of transcript abundance of *ALUMINUM ACTIVATED MALATE TRANSPORTER 9* (*MdALMT9*) in apple fruit in response to fruit load reduction, measured using quantitative RT-PCR. CC: Control fruit load-Cortex; CP: Control fruit load-Pith; RC: Reduced fruit load-Cortex; RP: Reduced fruit load-Pith. Fruit load reduction treatment was performed at 11 d after full bloom. The mean and S.E. of the mean (*n* = 4) are displayed. Asterisk indicates significant difference between control and reduced fruit load treatments in the cortex (α = 0.05). The transcript abundance data are presented in reference to mean expression at 0 d after treatment in RC. Transcript abundance was normalized to that of the apple *GAPDH* (*GLYCERALDEHYDE 3-PHOSPHATE DEHYDROGENASE*) gene. Shaded regions in the background indicate early (dark grey), mid (light grey), and late fruit development (white) periods.

**Additional file 2.** A correlation matrix among different fruit metabolites. Pearson correlation analysis was performed to determine relationships among all metabolites analyzed in this study. Fruit metabolite concentration data were used here. Correlations are presented as color-keyed correlation coefficients (numbers) and a heat map. Positive correlations are indicated in blue and negative correlations are in red. The intensity of the color represents the magnitude of the relationship between two metabolites.

**Additional file 3 **Spatiotemporal patterns of apple fruit tissue metabolite contents in response to fruit load reduction. Fruit metabolite concentration was multiplied with normalized tissue areas of the cortex and pith to obtain an estimate of tissue metabolite content. Tissue area was used as a proxy for tissue weight. The mean and standard error of the mean (*n* = 4) are displayed. CC: Control fruit load-Cortex; CP: Control fruit load-Pith; RC: Reduced fruit load-Cortex; RP: Reduced fruit load-Pith. Asterisks and dagger symbols indicate significant difference between control and reduced fruit load treatments in the cortex and pith, respectively (α = 0.05). Shaded regions indicate early (dark grey), mid (light grey), and late fruit development (white) periods.

**Additional file 4.** Fruit developmental stages of ‘Golden Delicious Smoothee’. Fruit developmental stages used in this study are displayed. Fruit represented here were longitudinally sliced and fixed in CRAF III fixative. Images were obtained using a flatbed scanner and processed with ImageJ. Bar indicates 1 cm. DAT: Days after treatment. Fruit load reduction was performed at 11 d after full bloom corresponding to 0 DAT.

**Additional file 5.** List of the apple genes and sequences of primers used in quantitative RT-PCR analyses.

**Additional file 6.** Statistical significance of fruit growth, metabolite concentration and transcript abundance data during fruit development, in cortex and pith tissues, and in response to fruit load reduction.

## Data Availability

Datasets used in the current study are available from the corresponding author on reasonable request.

## Abbreviations

CC: Control fruit load - Cortex
CL: Control fruit load
CP: Control fruit load - Pith
DAFB: Days after full bloom
DAT: Days after treatment
EFD: Early fruit development
F6P: Fructose-6-phosphate
Fru: Fructose
G1P: Glucose-1-phosphate
G6P: Glucose-6-phosphate
Glc: Glucose
Ino: Myo-inositol
LFD: Late fruit development
MFD: Mid fruit development
RC: Reduced fruit load - Cortex
RL: Reduced fruit load
RP: Reduced fruit load - Pith
Sor: Sorbitol
Suc: Sucrose
Xyl: Xylose
