# Supplementary material for: Higher growth of the apple (Malus × domestica Borkh.) fruit cortex is supported by resource intensive metabolism during early development
Source: BMC Plant Biol. 2020 Feb 13;20:75. doi: 10.1186/s12870-020-2280-2 (PMC7020378; doi:10.1186/s12870-020-2280-2)
Supplement: Supplementary file 1 — Additional file 1. Spatiotemporal patterns of transcript abundance of ALUMINUM ACTIVATED MALATE TRANSPORTER 9 (MdALMT9) in apple fruit in response to fruit load reduction, measured using quantitative RT-PCR. CC: Control fruit load-Cortex; CP: Control fruit load-Pith; RC: Reduced fruit load-Cortex; RP: Reduced fruit load-Pith. Fruit load reduction treatment was performed at 11 d after full bloom. The mean and S.E. of the mean (n = 4) are displayed. Asterisk indicates significant difference between control and reduced fruit load treatments in the cortex (α = 0.05). The transcript abundance data are presented in reference to mean expression at 0 d after treatment in RC. Transcript abundance was normalized to that of the apple GAPDH (GLYCERALDEHYDE 3-PHOSPHATE DEHYDROGENASE) gene. Shaded regions in the background indicate early (dark grey), mid (light grey), and late fruit development (white) periods. [file 12870_2020_2280_MOESM1_ESM.pdf]

Additional file 1.

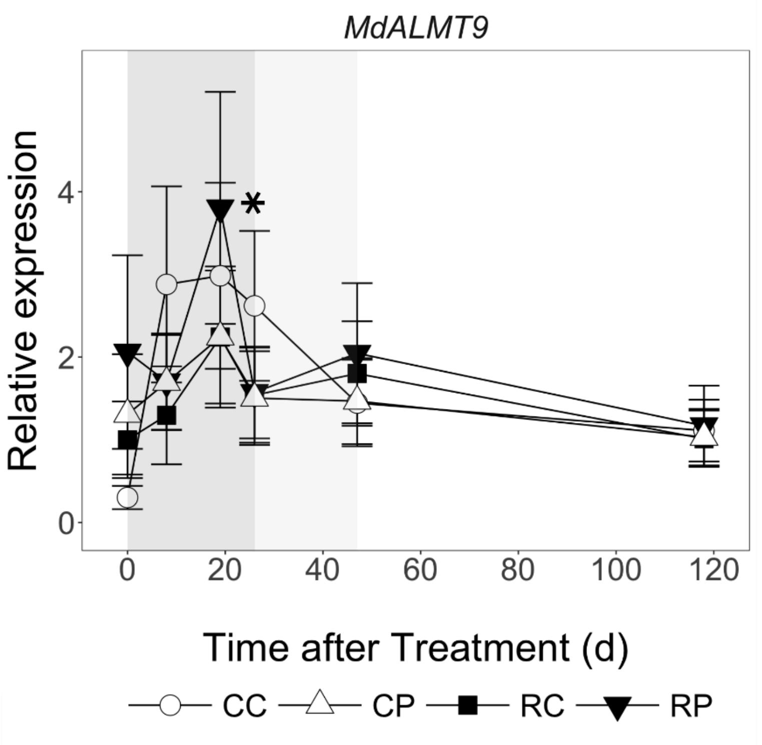

Additional file 1. Spatiotemporal patterns of transcript abundance of *ALUMINUM ACTIVATED MALATE TRANSPORTER 9* (*MdALMT9*) in apple fruit in response to fruit load reduction, measured using quantitative RT-PCR. CC: Control fruit load-Cortex; CP: Control fruit load-Pith; RC: Reduced fruit load-Cortex; RP: Reduced fruit load-Pith. Fruit load reduction treatment was performed at 11 d after full bloom. The mean and S.E. of the mean (n = 4) are displayed. Asterisk indicates significant difference between control and reduced fruit load treatments in the cortex (α = 0.05). The transcript abundance data are presented in reference to mean expression at 0 d after treatment in RC. Transcript abundance was normalized to that of the apple *GAPDH* (*GLYCERALDEHYDE 3-PHOSPHATE DEHYDROGENASE*) gene. Shaded regions in the background indicate early (dark grey), mid (light grey), and late fruit development (white) periods.
