# Supplementary material for: Higher growth of the apple (Malus × domestica Borkh.) fruit cortex is supported by resource intensive metabolism during early development
Source: BMC Plant Biol. 2020 Feb 13;20:75. doi: 10.1186/s12870-020-2280-2 (PMC7020378; doi:10.1186/s12870-020-2280-2)
Supplement: Supplementary file 2 — Additional file 2. A correlation matrix among different fruit metabolites. Pearson correlation analysis was performed to determine relationships among all metabolites analyzed in this study. Fruit metabolite concentration data were used here. Correlations are presented as color-keyed correlation coefficients (numbers) and a heat map. Positive correlations are indicated in blue and negative correlations are in red. The intensity of the color represents the magnitude of the relationship between two metabolites. [file 12870_2020_2280_MOESM2_ESM.pdf]

Additional file 2.

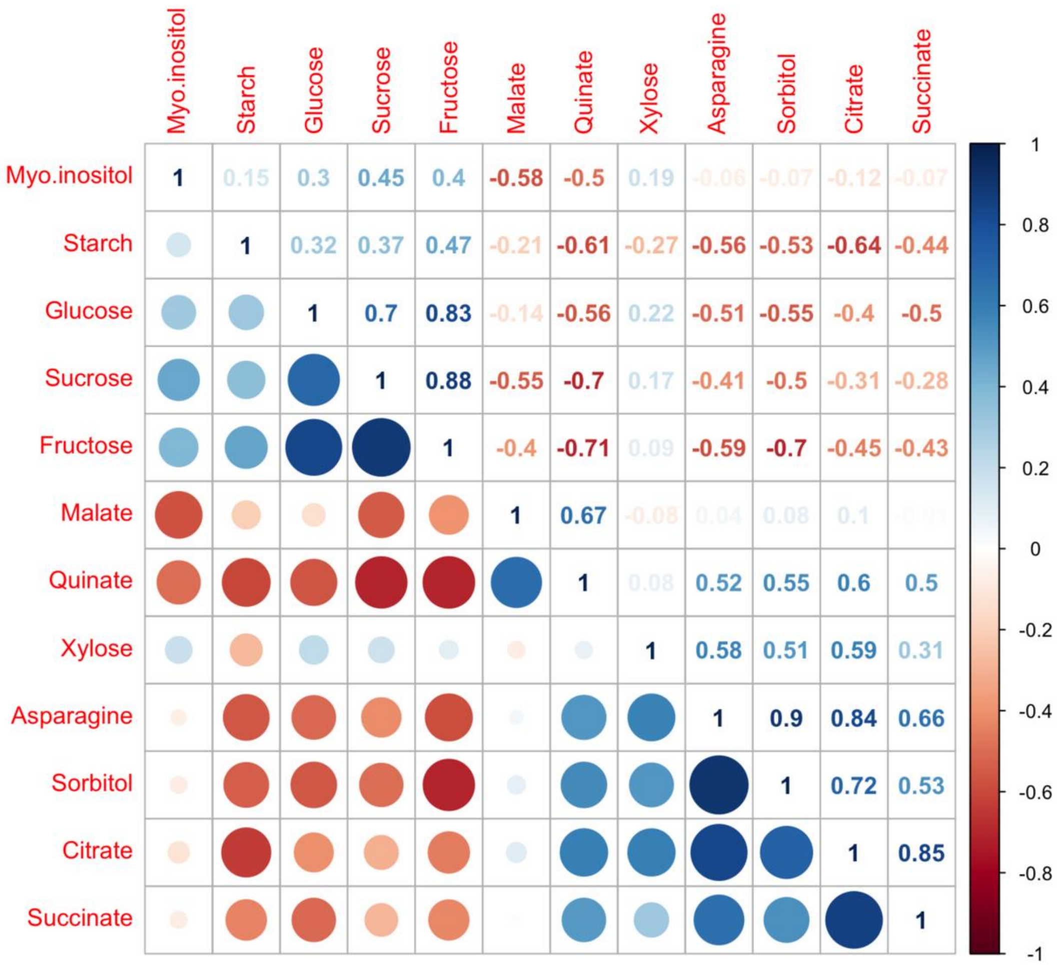

Additional file 2. A correlation matrix among different fruit metabolites. Pearson correlation analysis was performed to determine relationships among all metabolites analyzed in this study. Fruit metabolite concentration data were used here. Correlations are presented as color-keyed correlation coefficients (numbers) and a heat map. Positive correlations are indicated in blue and negative correlations are in red. The intensity of the color represents the magnitude of the relationship between two metabolites.
