# Supplementary material for: Higher growth of the apple (Malus × domestica Borkh.) fruit cortex is supported by resource intensive metabolism during early development
Source: BMC Plant Biol. 2020 Feb 13;20:75. doi: 10.1186/s12870-020-2280-2 (PMC7020378; doi:10.1186/s12870-020-2280-2)
Supplement: Supplementary file 3 — Additional file 3 Spatiotemporal patterns of apple fruit tissue metabolite contents in response to fruit load reduction. Fruit metabolite concentration was multiplied with normalized tissue areas of the cortex and pith to obtain an estimate of tissue metabolite content. Tissue area was used as a proxy for tissue weight. The mean and standard error of the mean (n = 4) are displayed. CC: Control fruit load-Cortex; CP: Control fruit load-Pith; RC: Reduced fruit load-Cortex; RP: Reduced fruit load-Pith. Asterisks and dagger symbols indicate significant difference between control and reduced fruit load treatments in the cortex and pith, respectively (α = 0.05). Shaded regions indicate early (dark grey), mid (light grey), and late fruit development (white) periods. [file 12870_2020_2280_MOESM3_ESM.pdf]

Additional file 3.

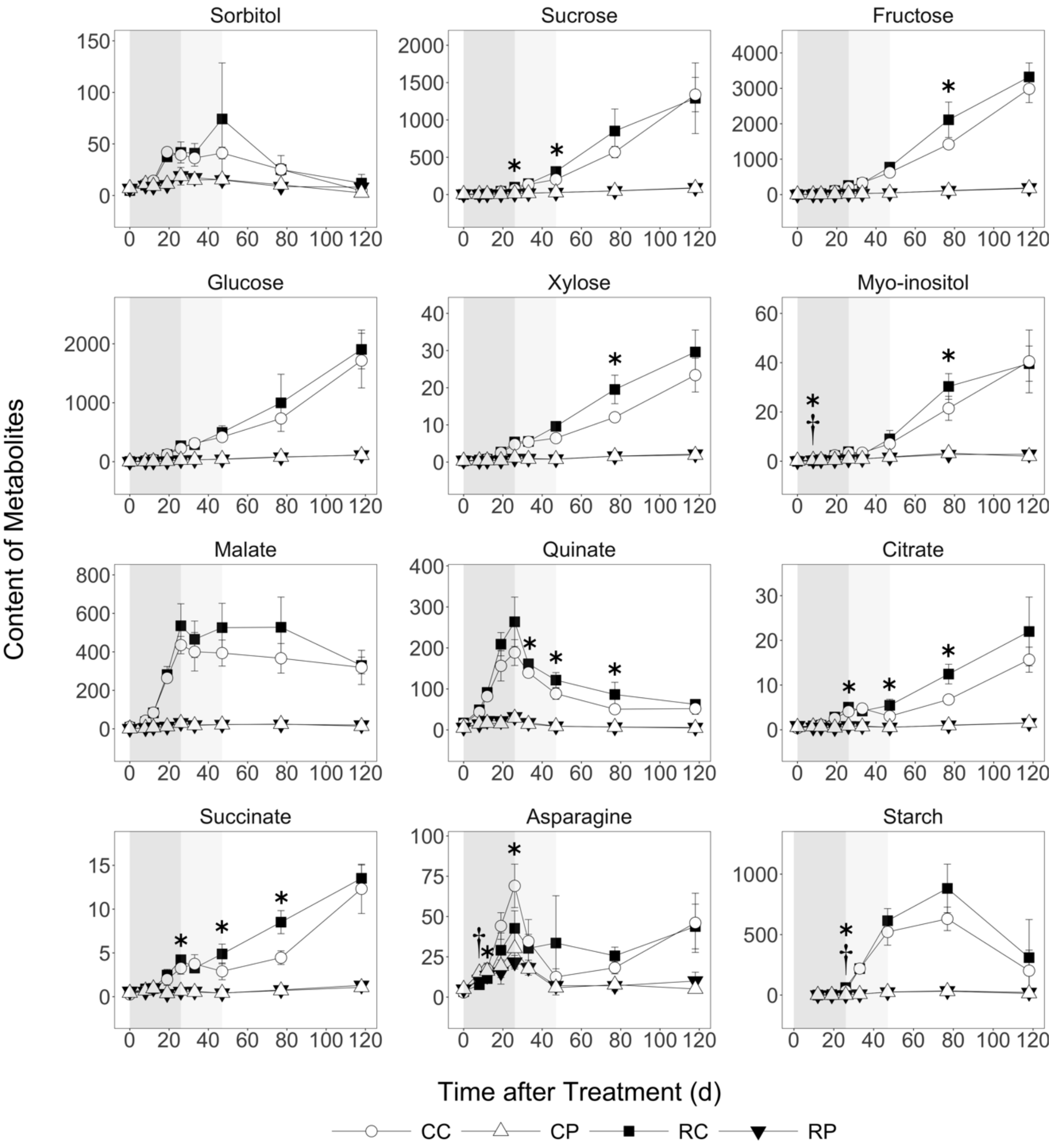

Additional file 3. Spatiotemporal patterns of apple fruit tissue metabolite contents in response to fruit load reduction. Fruit metabolite concentration was multiplied with normalized tissue areas of the cortex and pith to obtain an estimate of tissue metabolite content. Tissue area was used as a proxy for tissue weight. The mean and standard error of the mean (n = 4) are displayed. CC:

Control fruit load-Cortex; CP: Control fruit load-Pith; RC: Reduced fruit load-Cortex; RP: Reduced fruit load-Pith. Asterisks and dagger symbols indicate significant difference between control and reduced fruit load treatments in the cortex and pith, respectively ($\alpha = 0.05$). Shaded regions indicate early (dark grey), mid (light grey), and late fruit development (white) periods.
