# Supplementary material for: Higher growth of the apple (Malus × domestica Borkh.) fruit cortex is supported by resource intensive metabolism during early development
Source: BMC Plant Biol. 2020 Feb 13;20:75. doi: 10.1186/s12870-020-2280-2 (PMC7020378; doi:10.1186/s12870-020-2280-2)
Supplement: Supplementary file 4 — Additional file 4. Fruit developmental stages of ‘Golden Delicious Smoothee’. Fruit developmental stages used in this study are displayed. Fruit represented here were longitudinally sliced and fixed in CRAF III fixative. Images were obtained using a flatbed scanner and processed with ImageJ. Bar indicates 1 cm. DAT: Days after treatment. Fruit load reduction was performed at 11 d after full bloom corresponding to 0 DAT. [file 12870_2020_2280_MOESM4_ESM.pdf]

Additional file 4.

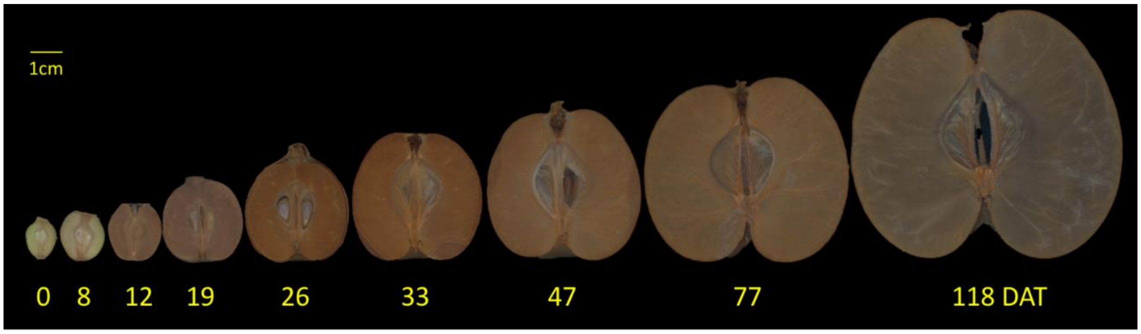

Additional file 4. Fruit developmental stages of 'Golden Delicious Smoothee'. Fruit developmental stages used in this study are displayed. Fruit represented here were longitudinally sliced and fixed in CRAF III fixative. Images were obtained using a flatbed scanner and processed with ImageJ. Bar indicates 1 cm. DAT: Days after treatment. Fruit load reduction was performed at 11 d after full bloom corresponding to 0 DAT.
