# Supplementary material for: Higher growth of the apple (Malus × domestica Borkh.) fruit cortex is supported by resource intensive metabolism during early development
Source: BMC Plant Biol. 2020 Feb 13;20:75. doi: 10.1186/s12870-020-2280-2 (PMC7020378; doi:10.1186/s12870-020-2280-2)
Supplement: Supplementary file 5 — Additional file 5. List of the apple genes and sequences of primers used in quantitative RT-PCR analyses. [file 12870_2020_2280_MOESM5_ESM.pdf]

Additional file 5. List of the apple genes and the sequence of primers used in quantitative RT-PCR analyses.

Gene	Accession Number	Primer Orientation	Primer Sequence (5'-3')
MdACTIN	EB127077	Forward	ACCATCTGCAACTCATCCGAACCT
		Reverse	ACAATGCTAGGGAACACGGCTCTT
MdGAPDH	EB146750	Forward	TGAGGGCAAGCTGAAGGGTATCTT
		Reverse	TCAAGTCAACCACACGGGTACTGT
MdSDH1	MDP0000932467	Forward	GAGTCTTGGCGCAGATGCAGT
		Reverse	ACAGTCGAAGGTTACATCCACTCCATT
MdSDH2	MDP0000874667	Forward	CATTGCCAGCAGTGCAAAGGC
		Reverse	GGCAATTTAAAGCACAGATCCGCG
MdSDH5	MDP0000250546	Forward	GTTAGAGATGTCAAACCTGTGGAGA
		Reverse	GCAAATGCCGACAGCCTTAATT
MdSDH9	MDP0000188052	Forward	CCTGCAATGGCATGGTTAGACAA
		Reverse	CACAAATGCCGACAGCCTTG
MdCwINV	MDP0000275150	Forward	CCTCATCAATTGGGAAGCTCTTGAG
		Reverse	GATAGGGGTCCGACGCATTTTTC
MdNINV3	MDP0000186866	Forward	GTACTCCATGATCCTGTCCGGAATAGT
		Reverse	CATACCCTTCTGGCATTCAGGCAG
MdNINV4	MDP0000652278	Forward	GTCTTGGCCAATCTGGGATAATAT
		Reverse	ATTCACACGGGTCTCAATTGAC
MdNINV6	MDP0000261740	Forward	AATGCCCAATTGTGTACAAATGCG
		Reverse	GGTATCGCTTTATCTGATTCGTCTACA
MdVINV3	MDP0000377084	Forward	CCCTGACGGCCAAATCATAATGT
		Reverse	GAAATCAGTGGATCCGATCCCG
MdSUSY3	MDP0000126946	Forward	GGAAAAGAATACTGCAGCCGCACG
		Reverse	GAACTTCGCTGAAAGGTCCGGT

MdSPS2	MDP0000288684	Forward	GCATCACAAGCAATCAGATGTACCT
		Reverse	CAAGCCACAGGTTTTTGTCTCCT
MdSPS3	MDP0000331376	Forward	CGAGGGAGAGAAGGGAGATTTG
		Reverse	TCTGCTGACTAATCCATGTCTCCATT
MdFK1	MDP0000173131	Forward	GACTGGTGGTGATGATCCTTGC
		Reverse	CCCGCCCATGGAATTTCTGT
MdFK3	MDP0000309723	Forward	AAGCATTTGCAGGAGATGTGCTAT
		Reverse	GCTTCAGCCAATGAAAGTCCATTAGTA
MdFK4	MDP0000765663	Forward	TCTAGCTGCTATGAAAATTGCCAAGG
		Reverse	CCAGTCAGGAATCTAATTTCATCCTCG
MdHXK3	MDP0000643891	Forward	GGCAAGATGTAGTGGCTGAATTG
		Reverse	CCTCCAGCTAATGTCCCAACC
MdAGPase3	MDP0000203812	Forward	AACTCTTCATCCAAGTTGGCCAG
		Reverse	GATTATACTGCTGGAAATGCATGCA
MdAGPase4	MDP0000394192	Forward	GAAACGTTCTGGTGGACGAGACT
		Reverse	ATGCTGCTGGAAATGCAAGCG
MdAGPase5	MDP0000323050	Forward	GAGGCTCACAAAAAATATGGTGGG
		Reverse	AGTGGAAACACCTTCAACGGC
MdSBE2	MDP0000214735	Forward	AAACAATGCAGATGGCTCACCTTC
		Reverse	AACATGTGCCTCATAAATGCGAAGTG
MdSS1	MDP0000842179	Forward	GACTGACAGCTCTGTGCTTGTTC
		Reverse	GAGCTCTTTCCAATCGCATGAAG
MdPEPC1	MD03G1242000	Forward	CTCAAAGCGAAAACCTAGTGGTGGT
		Reverse	GCTCCAAGGCCTAACCACACA
MdPEPC2	MD17G1230800	Forward	TCCAAATGCTTCGGGAGATGTATAATCAG
		Reverse	CTGACACGAGAAGCTTGTCATACAGAGAG
MdMDH2	MD07G1073300	Forward	ACTCTCACCGCCGTGTGG
		Reverse	AACACGCCGAAGCACTCAGG
MdMDH4	MD16G1219000	Forward	GGCCTTGGGCCAGGTTTCT
		Reverse	ACAAGCTCACGGACACCTTTCTCC

MdASPA1	MD06G1205500	Forward	GCTAGGGAACAGGGTGTTGAGACT
		Reverse	TCACCATCAGCATCTGGTGTTTCTTCT
MdASPA4	MD08G1092600	Forward	AGGCGTAGAGAGTCCGTTGGT
		Reverse	CAGCGCAACGCCCTTGGC
MdGS1	MD17G1268700	Forward	CATCAACCTGGATCTCTCAGGCTCT
		Reverse	TTCACTATCTTCTCCAGGAGCTTGACC
MdGS3	MD13G1180400	Forward	GCAGATGAAGATTACAAGGAGCTCAACC
		Reverse	GTTCAGTAAACCCTCTAGCCTGTTGATT
MdALMT9	MDP0000252114	Forward	TATTGCTGTCGGTGCCGCCAC
		Reverse	ACTGATTAACAACACCTTCCAAAGAAGC
